# Dual Proteomics Strategies to Dissect and Quantify the Components of Nine Medically Important African Snake Venoms

**DOI:** 10.3390/toxins17050243

**Published:** 2025-05-13

**Authors:** Damien Redureau, Fernanda Gobbi Amorim, Thomas Crasset, Imre Berger, Christiane Schaffitzel, Stefanie Kate Menzies, Nicholas R. Casewell, Loïc Quinton

**Affiliations:** 1 Laboratory of Mass Spectrometry, MolSys Research Unit, University of Liège, B4000 Liège, Belgium; dredureau@uliege.be (D.R.); thomas.crasset@uliege.be (T.C.); 2 School of Biochemistry, University of Bristol, 1 Tankard’s Close, Bristol BS8 1TD, UK; imre.berger@bristol.ac.uk (I.B.); christiane.berger-schaffitzel@bristol.ac.uk (C.S.); 3 Max Planck Bristol Centre for Minimal Biology, Cantock’s Close, Bristol BS8 1TS, UK; 4 Centre for Snakebite Research & Interventions, Department of Tropical Disease Biology, Liverpool School of Tropical Medicine, Liverpool L3 5QA, UKnicholas.casewell@lstmed.ac.uk (N.R.C.); 5 Department of Biomedical and Life Sciences, Lancaster University, Lancaster LA1 4YG, UK

**Keywords:** MELD, trypsin, *Echis*, *Dendroaspis*, shotgun proteomic, Elapidae, Viperidae

## Abstract

Snakebite envenoming constitutes a significant global health issue, particularly in Africa, where venomous species such as *Echis* vipers and *Dendroaspis* mambas pose substantial risks to human health. This study employs a standardized venomics workflow to comprehensively characterize and comparatively quantify the venom composition of nine medically relevant snake species chosen from among the deadliest in Africa. Utilizing shotgun venom proteomics and venom gland transcriptomics, we report detailed profiles of venom complexity, highlighting the relative abundance of dominant toxin families such as three-finger toxins and Kunitz-type proteins in *Dendroaspis*, and metalloproteinases and phospholipases A_2_ in *Echis*. We delineate here the relative abundance and structural diversity of venom components. Key to our proteomic approach is the implementation of Multi-Enzymatic Limited Digestion (MELD), which improved protein sequence coverage and enabled the identification of rare toxin families such as hyaluronidases and renin-like proteases, by multiplying the overlap of generated peptides and enhancing the characterization of both toxin and non-toxin components within the venoms. The culmination of these efforts resulted in the construction of a detailed toxin database, providing insights into the biological roles and potential therapeutic targets of venom proteins and peptides. The findings here compellingly validate the MELD technique, reinforcing its reproducibility as a valuable characterization approach applied to venomics. This research significantly advances our understanding of venom complexity in African snake species, including representatives of both Viperidae and Elapidae families. By elucidating venom composition and toxin profiles, our study paves the way for the development of targeted therapies aimed at mitigating the morbidity and mortality associated with snakebite envenoming globally.

## 1. Introduction

Snakebites pose a significant global public health challenge, with particularly high prevalence in regions such as the Middle East, Asia, Africa, and other tropical and subtropical areas [1]. According to the World Health Organization (WHO), approximately 5.4 million snakebite incidents are reported annually, resulting in approximately 2.7 million instances of envenoming, leading to ~138,000 fatalities and up to three times more disabilities, including amputations [2,3,4]. These alarming data underline the urgent need for effective snakebite management strategies to mitigate the associated morbidity and mortality. Achieving this goal necessitates a precise molecular-scale characterization of venom composition, as this understanding is fundamental to improving snakebite management. By identifying key toxin families and their functional roles, this knowledge enables the development of targeted antivenoms, enhances treatment efficacy, and informs therapeutic strategies to mitigate snake envenoming effects more effectively.

Snake venoms are complex biochemical mixtures, composed primarily of proteins and peptides, which account for over 90% of the venom dry weight [5]. The toxicity of snake venoms is mainly attributed to a diverse array of toxins, which are expressed at different concentrations in different venoms [6]. These toxins encompass various categories, such as neurotoxins, hemotoxins [7], cytotoxins, myotoxins [8] and others, often exerting synergistic effects. Key families of toxins found in snake venoms include phospholipases A_2_ (PLA_2_s), L-amino acid oxidases (LAAOs), snake venom metalloproteinases (SVMPs), snake venom serine proteases (SVSPs), C-type lectins, disintegrins, Kunitz-type toxins (vKunitz-type) and three-finger toxins (3FTxs). Each of these toxin families contributes uniquely to the intricate pathophysiological manifestations observed following snakebite envenoming [6]. Snakes of the species-rich *Echis* genus, commonly known as saw-scaled vipers or carpet vipers, are widely distributed across Africa, the Middle East, and parts of Asia, where they thrive in diverse environments ranging from arid deserts to grassy savannas [9]. Species within the *Echis* genus display unique adaptations, such as their ability to produce a distinctive rasping sound by rubbing their serrated scales together, a behavior used to deter predators. These vipers, commonly encountered in rural and agricultural settings, cause severe effects in humans following envenomation. Symptoms include blistering, edema, progressive necrosis around the bite site, and life-threatening systemic effects such as hemorrhage, coagulopathy, and occasionally hypovolemic shock [10,11,12]. Notably, many of the toxic effects, particularly those affecting the circulatory system, are classified as hemotoxic and are primarily driven by enzymes such as snake venom metalloproteinases (SVMPs), which induce venom-induced consumption coagulopathy (VICC), severe hemorrhage, and local tissue damage. These effects make *Echis* bites particularly problematic and dangerous, and the rapid progression of symptoms necessitates prompt medical attention to mitigate potentially fatal outcomes. In contrast, the genus *Dendroaspis* includes only four recognized species: *D. polylepis*, *D. angusticeps*, *D. viridis* and *D. jamesoni*, the latter of which comprises two subspecies: *D. jamesoni jamesoni* and *D. jamesoni kaimosae* [9]. Each species displays unique adaptations and venom profiles, reflecting their distinct ecological niches and geographical distributions across Africa. For example, the black mamba *D. polylepis* inhabits diverse habitats from savannas to dense forests. In contrast, green mambas are predominantly arboreal, thriving in forested regions and coastal areas. In contrast to *Echis* venom, the venom of *Dendroaspis* species is predominantly neurotoxic, targeting the nervous system with components like dendrotoxins and calciseptine, which are members of the Kunitz and 3FTxs family. Members of these toxin families interfere with ion channels and neurotransmitter release, leading to rapid paralysis and respiratory failure in envenomed prey or victims [13,14,15,16]. Despite the lethal potential of their venoms, mambas generally avoid humans, though defensive bites present significant medical challenges due to the rapid onset of symptoms and potential mortality if untreated.

Antivenom remains the cornerstone treatment for snakebites, crucial for neutralizing the toxic effects of snake venoms and mitigating systemic complications [17]. Current antivenoms are typically derived from the serum of venom-hyperimmunized animals and consist of purified immunoglobulins G (IgG) or fragmented forms like F(ab) or F(ab’)_2_. Despite the efficacy of such products, which save thousands of lives every year, antivenoms face several limitations [18,19,20]. For example, their administration presents challenges due to potential adverse effects that can exacerbate patients’ conditions. These adverse reactions include acute responses like nausea, fever, and more severe immune reactions such as anaphylaxis and hypotension. Pyrogenic reactions, characterized by fever, tachycardia, and vasodilation, are also attributed to antivenoms, while delayed reactions may manifest as fever, joint pain, skin eruptions, and lymphadenopathy. Venom immunization protocols have also not evolved significantly over the past century, relying on whole venom to stimulate immune responses and lacking specificity towards the most pathogenic venom proteins. Consequently, antivenom efficacy may be compromised by the production of redundant antibodies targeting non-toxic molecules and a deficiency in potent neutralizing antibodies against small, weakly immunogenic toxins. To overcome these challenges, several innovative approaches are being explored. These include virus-like particle (VLP)-based antivenoms, such as those developed in the ADDovenom project (EU Horizon 2020) [21], as well as recombinant human or humanized monoclonal antibodies [22] and small-molecule inhibitors like batimastat, marimastat (SVMP inhibitors) [23,24,25], and varespladib (PLA_2_ inhibitor) [26]. Recent breakthroughs also include AI-designed protein binders capable of neutralizing 3FTxs, a major class of elapid toxins, in both in vitro and in vivo models [27].

In response to the critical challenges posed by snakebite envenomation, venomics [28] and antivenomics [28] have emerged as indispensable tools for enhancing our understanding of venom composition, toxin–antivenom interactions, and the development of more effective and targeted antivenoms via target identification for toxin neutralization. *Venomics*, as an integrative proteotranscriptomics approach, provides a comprehensive view of venom complexity, revealing the molecular composition and functional diversity of venom components. This diversity is influenced by numerous factors, including gender, age, and geographical location of the snakes, all of which significantly impact venom potency and lethality [29]. Understanding this variability is essential for improving the specificity and efficacy of antivenoms and for tailoring treatments to different envenomation contexts, such as variations in venom toxicity between populations of the same species, differing clinical symptoms based on toxin dominance, or regional differences in available medical care and antivenom accessibility. Addressing these factors ensures that antivenoms are optimized for geographically distinct venoms and variable clinical presentations, ultimately improving treatment outcomes across diverse snakebite scenarios.

This study focuses on venoms from nine of the most medically significant snake species in Africa. These include the five species and subspecies of the genus *Dendroaspis* (*D. polylepis*, *D. angusticeps*, *D. viridis*, *D. j. jamesoni* and *D. j. kaimosae*), as well as four species of *Echis* (*E. leucogaster*, *E. pyramidum leakeyi*, *E. coloratus*, and *E. romani*). Cladograms for both genera are presented in Figure S1. These snakes represent a wide range of venom phenotypes and toxicological profiles, making them ideal for advancing venom research. The methodological framework integrates advanced proteomics and transcriptomics, incorporating classical sodium dodecyl sulfate polyacrylamide gel electrophoresis (SDS-PAGE), a well-established and foundational technique for protein separation, alongside modern liquid chromatography–mass spectrometry (LC-MS/MS) for detailed proteomic analysis. While SDS-PAGE serves as a preliminary step to ensure the quality and integrity of protein samples, the strength of this study lies in the combination of MELD (Multi-Enzymatic Limited Digestion) with LC-MS/MS, supported by robust bioinformatics pipelines [30,31]. Unlike traditional single-enzyme digestion methods, MELD was specifically designed to address the complexity of protein mixtures, like snake venoms, which contain multiple enzymatic cleavage sites and highly diverse isoforms. In MELD, each mixture of proteases (trypsin, GluC, chymotrypsin) is used in parallel at two concentration levels to digest separate aliquots of the same sample under controlled conditions. These digests are then pooled prior to LC-MS/MS to generate a peptide pool enriched in overlapping, enzyme-specific fragments. This limited yet complementary digestion enhances proteome coverage without overloading the sample’s complexity and enables improved peptide–spectrum matching. As demonstrated in the original MELD method development study [30], this approach is particularly effective for complex matrices, where classical trypsin-only protocols often fall short. Following digestion, high-resolution LC-MS/MS allows for the reconstruction of full-length proteins through the alignment of redundant overlapping peptides, improving the detection of isoforms, post-translational modifications, and domain-level features. This approach facilitates a more comprehensive understanding of venom complexity by enabling precise mapping of venom toxins, including the identification of major toxin families [31].

Finally, a classical bottom–up proteomics approach using trypsin only was strategically employed in this study to complement MELD. This decision was made to streamline the identification of highly abundant venom proteins and to ensure compatibility with downstream LC-MS/MS analyses. This step, while traditional, was critical in establishing a baseline proteome profile, which was further enriched by the insights gained from MELD. Additionally, tryptic digestion facilitates a semi-quantitative approach by leveraging the relative intensity of the ion chromatographic areas corresponding to peptides unique to a given protein [32,33] by targeting the sum of unique peptides areas generated by tryptic digestion. In the context of this study, trypsin-based digestion provides a baseline quantification of highly abundant proteins, which is critical for understanding the relative contribution of each toxin to the overall venom composition. Collectively, this dual strategy of enhanced proteomic resolution (MELD) and semi-quantitative assessment (trypsin only) resulted in detailed characterization and inter-specific comparisons of venom complexity in medically important African snakes and lays the groundwork for long-term improvement of snakebite therapy via the discovery of targeted toxin-specific inhibitory molecules. The goal of the present study is to comprehensively characterize the venoms of key African snake species relevant to envenomation and antivenom development. The resulting data will contribute to a dedicated database of major toxins, which will be made publicly available on the ADDovenom website (https://www.addovenom.com, accessed on 1 May 2025).

## 2. Results

### 2.1. Complexity and Diversity of Echis and Dendroaspis Venoms

**Figure 1:**
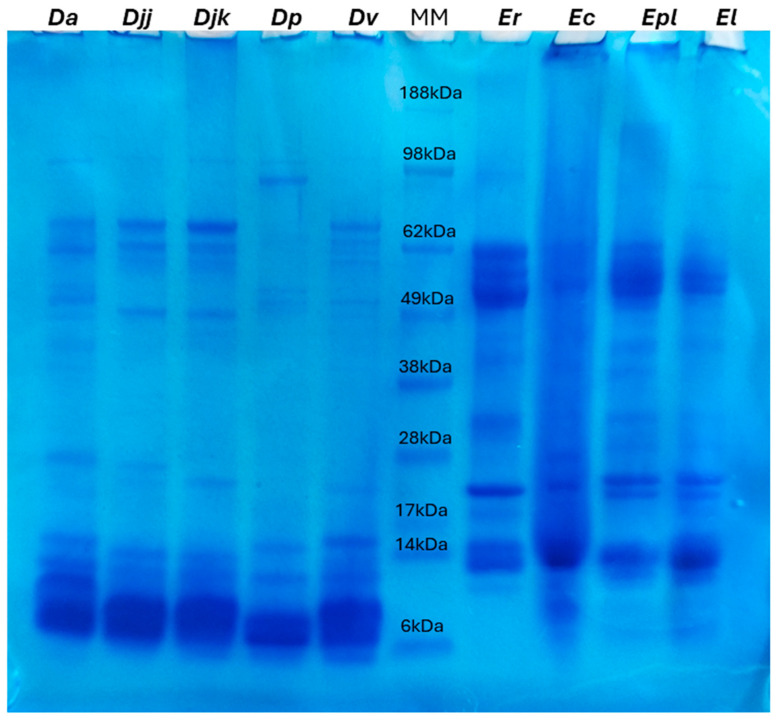
SDS-PAGE sections of the nine snake venoms studied, obtained in reducing/denaturing conditions. The SDS-PAGE gel displays protein profiles from nine venom samples (30 μg each) belonging to the genera *Dendroaspis* (left, five species) and *Echis* (right, four species), alongside molecular weight markers (MM) for reference (6 μg). Protein molecular weights range from 6 kDa to 188 kDa, allowing for the identification of protein bands corresponding to key venom components. Columns *Da* and *Djj*: venoms from *D. angusticeps* and *D. j. jamesoni*, respectively. Columns *Djk* and *Dp*: venoms from *D. j. kaimosae* and *D. polylepis*. Column *Dv*: venom from *D. viridis.* Columns *Er* and *Ec*: venoms from *E. romani* and *E. coloratus*. Column *Epl* and *El*: venoms from *E. p. leakeyi* and *E. leucogaster*.

The SDS-PAGE analysis revealed distinct protein profiles for venoms from the nine snake species, with *Dendroaspis* venoms shown in the left panel and *Echis* venoms in the right panel of Figure 1 (and Figure S2, for its uncropped version). A duplicate of this SDS-PAGE analysis is available for the five *Dendroapsis* in Figure S3 and for the four *Echis* in Figure S4. Protein molecular weights, ranging from 6 kDa to 188 kDa, were used to infer the identity and abundance of key venom components. In *Dendroaspis* venoms, prominent protein bands were observed in the low-molecular-mass range (6–14 kDa), consistent with the presence of 3FTxs and vKunitz-type, which are hallmark neurotoxins of mamba venoms. Faint bands in the mid-molecular-mass range (28–49 kDa) suggest the presence of less abundant proteins, such as SVMPs and serine proteases (SVSPs). Notably, *D. polylepis* exhibited a distinct high-molecular-mass band at approximately 98 kDa, suggesting the potential presence of SVMP isoforms or other high-molecular-weight venom proteins. Similar bands, though varying in intensity, were also observed in other *Dendroaspis* venoms between 62 and 98 kDa, indicating that high-molecular-weight components, such as other SVMPs, may be a shared feature among these species. In contrast, *Echis* venoms displayed a dominance of high-molecular-mass bands (49–62 kDa) that could correspond to hemotoxic components, including SVMP but also hyaluronidases and LAAOs. Additionally, mid-molecular-mass bands (28–38 kDa) were present in *Echis* species, likely representing other members of the SVMP family or SVSP. The low-molecular-mass range (6–17 kDa) was less prominent in *Echis* venoms compared to *Dendroaspis*, although *E. leucogaster* exhibited intense bands in this range, reflecting an elevated concentration of possibly PLA_2_s and/or C-type lectin-like proteins.

The MALDI-TOF MS spectra of *Dendroaspis* venoms (Figure S5) revealed distinct patterns across different *m*/*z* ranges, reflecting variations in venom complexity. In the low-*m*/*z* range (600–10,000), the spectra exhibited a high number of peaks within *m*/*z* 6000–8500, suggesting a predominance of lower-mass venom components. The MALDI-TOF MS analysis of *Echis* venoms (Figure S6) revealed a broad distribution of peaks across different *m*/*z* ranges, reflecting varying venom complexity. In the low-*m*/*z* range (1,000–25,000), the spectra showed a high number of peaks concentrated between *m*/*z* 5,000 and 15,000, suggesting the presence of multiple low-molecular-mass venom components. In the high-*m*/*z* range (20,000–100,000), the spectra exhibited numerous peaks in the *m*/*z* 30,000–50,000 region. These ranges correspond broadly to well-characterized venom components, such as short neurotoxins (Figure S5) and high-mass enzymes like SVMPs (Figure S6). For instance, a strong signal at 7.9 kDa in the *D. polylepis* spectrum (Figure S5) is consistent with the expected mass of Kunitz-type protease inhibitors—among the most abundant toxins in this species—while other *Dendroaspis* venoms show more distributed peaks between ~6.3 and 7.2 kDa (Figure S5), aligning with diverse 3FTx isoforms. A distinct 8.3 kDa signal in *Dendroaspis* venoms, absent in *D. polylepis*, supports proteomic findings of interspecies differences in toxin composition. Overall, the number and distribution of peaks in the MALDI-TOF MS spectra correlate with the SDS-PAGE findings, where venoms with more intense peaks in specific *m*/*z* ranges also displayed stronger bands at corresponding molecular weights.

### 2.2. Proteomics Characterization of Dendroaspis Venoms

Table 1 presents the results gathered for the five mamba venoms using both trypsin and MELD digestion protocols. It showcases the number of peptide–spectrum matches (PSMs), the number of identified peptides, the number of identified proteins (built from the previous identified peptides), and the number of top proteins (i.e., the proteins which are supported with the greatest number of unique peptides among the identified proteins) for each species. These data were obtained from the database search and de novo sequencing against species-specific venom gland transcriptomes. *D. j. jamesoni* exhibited the highest number of peptide–spectrum matches under MELD digestion (7315) and the most identified proteins (158), with 110 classified as top proteins. *D. viridis* also displayed high protein diversity, with the greatest number of total (187) and top proteins (136), along with 5589 PSMs under MELD. In contrast, *D. polylepis* showed the lowest peptide–spectrum matches (2932) and the fewest peptides (951) under MELD protocol, indicating the least complex venom composition. *D. j. kaimosae* exhibited lower values in all categories compared to the other species, with 4757 peptide–spectrum matches, 1240 peptides, and 101 total proteins under MELD. *D. angusticeps* had a comparable number of peptide–spectrum matches (5513) to *D. viridis*, but with fewer total proteins (137) and top proteins (90). The results obtained from the trypsin digestion show a consistent trend with fewer identifications across all metrics, validating the added depth provided by the MELD strategy. Overall, *D. j. jamesoni* and *D. viridis* exhibited the highest venom diversity in terms of peptide–spectrum matches and protein numbers, while *D. polylepis* displayed the lowest complexity across all measured parameters in the MELD protocol approach. Importantly, the MELD results were used for comprehensive venom characterization, while trypsin-based digestion enabled relative quantification through peptide-area-based methods. This dual strategy maximized both depth and quantification accuracy in the analysis.

Venom components were classified into four major categories: toxins, non-toxins, cellular components, and unassigned proteins. The toxin group includes protein families well documented for their direct role in envenomation pathology, such as 3FTxs, SVMPs, SVSPs, PLA_2_s, venom Kunitz-type protein, and natriuretic peptides. The non-toxin category encompasses venom components that, while not directly cytotoxic or enzymatically active in classic envenomation symptoms, contribute to venom spread or modulation of host responses. These include aminopeptidases, 5′-nucleotidases, prokineticins, phosphodiesterases, venom endothelial growth factors (VEGFs), cathepsins, cysteine-rich venom proteins (CRISPs), waprins, cystatins, and hyaluronidases. Two additional families were used to classify the detected proteins: cellular components which group the proteins classically found in different parts of the cell (i.e., cytoplasm, nucleus, membrane, etc.) typically derived from venom gland cellular turnover or cells damaged during extraction, and unassigned proteins where no clear identification could be attributed to the sequence.

**Figure 2:**
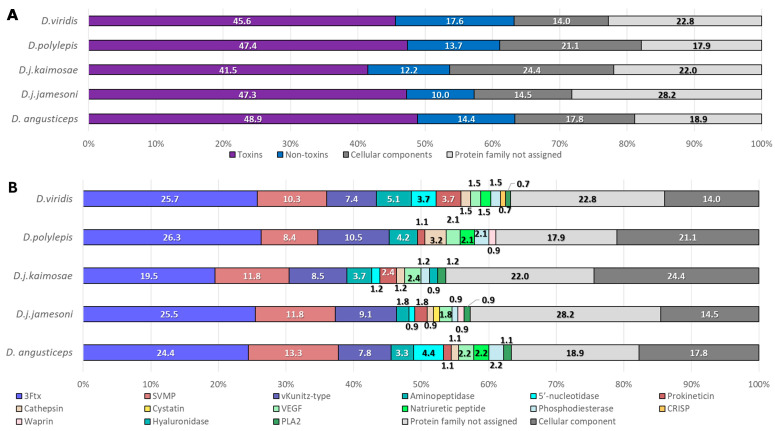
(**A**) Overview of the composition of toxins, non-toxins, cellular components, and unassigned protein families for each *Dendroaspis* venom. (**B**) Detailed breakdown of venom component classes for each species, highlighting the most prevalent classes such as three-finger toxins, vKunitz-type family, and SVMPs.

The distribution of the proteins within these four families varied across the five *Dendroaspis* venoms (Figure 2A). Protein sequences matching toxins constituted 40% to 50% of the total number of protein sequences in the five venom samples. Among them, *D. angusticeps* venom (*Da*) exhibited the highest toxin proportion at 48.9% (44 sequences), followed by *D. j. jamesoni* (*Djj*) at 47.3% (52 sequences) and *D. polylepis* (*Dp*) at 47.4% (45 sequences). *D. j. kaimosae* (*Djk*) had the lowest toxin proportion at 41.5% (34 sequences), while *D. viridis* (*Dv*) was slightly higher at 45.6% (62 sequences). Non-toxins were most abundant in *Dv* at 17.6% (24 sequences), while *Djj* displayed the lowest proportion at 10.0% (11 sequences). *Da*, *Djk*, and *Dp* exhibited intermediate levels at 14.4% (13 sequences), 12.2% (10 sequences), and 13.7% (13 sequences), respectively. Cellular components showed considerable variability, peaking at 24.4% (20 sequences) in *Djk* and 24.1% (23 sequences) in *Dp*, while *Da*, *Djj*, and *Dv* had 17.8% (16 sequences), 14.5% (16 sequences), and 14.0% (19 sequences), respectively. The relative importance of these findings remains unclear and could simply reflect natural biological variation in the collected samples. The unassigned protein families’ category was most prevalent in *Djj* at 28.2% (31 sequences), with *Da* at the lowest proportion of 18.9% (17 sequences). *Djk*, *Dp*, and *Dv* contained intermediate levels at 22.0% (18 sequences), 17.9% (17 sequences), and 22.8% (31 sequences), respectively.

Regarding specific toxin families (Figure 2B), 3FTxs were the most represented across all samples. *Dp* exhibited the highest proportion of 3FTxs at 26.3% (25 sequences), followed by *Dv* at 25.7% (35 sequences) and *Djj* at 25.5% (29 sequences). *Da* and *Djk* showed slightly lower levels at 24.4% (22 sequences) and 19.5% (16 sequences), respectively. vKunitz-type proteins were most prominent in *Dp* at 10.5% (10 sequences), with *Djj* at 9.1% (10 sequences) and *Djk* at 8.5% (seven sequences). *Da* and *Dv* had comparable levels at 7.8% (seven sequences) and 7.4% (10 sequences), respectively. SVMP were most abundant in *Da* at 13.3% (12 sequences), with *Djj* and *Djk* following at 11.8% (13 sequences) and 11.0% (nine sequences), respectively, while *Dv* (10.3%, 14 sequences) and *Dp* (8.4%, eight sequences) exhibited the lowest levels.

Among non-toxins, aminopeptidases were most detected in *Dv* at 5.1% (seven sequences), followed by *Dp* at 4.2% (four sequences), *Djk* at 3.7% (three sequences), and *Da* at 3.3% (three sequences). *Djj* contained the lowest level (1.8%, two sequences). Cathepsins varied significantly, peaking in *Dp* at 3.2% (three sequences) and reaching the lowest levels in *Djj* (0.9%, one sequence). Other toxins, such as natriuretic peptides and phosphodiesterases, were found in *Da* (2.2%, two sequences) and *Dp* (2.1%, two sequences) but were not detected in *Djj* and *Djk*. Venom endothelial growth factors ranged between 1.5% (two sequences) and 2.4% (three sequences) across all samples. Natriuretic peptides were found exclusively in *Da*, *Dv*, and *Dp*, while PLA_2_s was not detected in *Dp*. Hyaluronidases were uniquely detected in *Djk*. Cysteine-rich venom proteins were more abundant in *Dv* (2.1%, three sequences), waprins in *Dp* (1.1%, one sequence), and cystatins in *Djj* (0.9%, one sequence). These findings highlight significant interspecies variations in venom composition, which may contribute to differences in toxicological effects and clinical outcomes.

### 2.3. Proteomics Characterization of Echis Venoms

Table 2 presents the results gathered for *Echis* venoms, analyzed using both trypsin and MELD digestion protocols. The venom composition of the four species demonstrated substantial variability in terms of peptide–spectrum matches, unique peptides, and identified proteins. *E. coloratus* exhibited the highest number of peptide–spectrum matches (14,632), the most identified peptides (4126), and the greatest number of total proteins (144), with 121 classified as top proteins using MELD. In contrast, *E. romani* showed a relatively high number of peptide–spectrum matches (10,377) and identified peptides (3104), with 124 total proteins and 96 top proteins using MELD. *E. leucogaster* displayed 9461 peptide–spectrum matches and 2584 identified peptides, with a total of 140 proteins, of which 89 were top proteins using MELD. *E. pyramidum leakeyi* had a similar number of peptide–spectrum matches (9364) but identified more peptides (3007) than *E. leucogaster*, with 136 total proteins and the lowest number of top proteins (87) among the four species. Overall, *E. coloratus* exhibited the highest venom complexity across all parameters, while *E. romani* showed high diversity in peptides and proteins. *E. leucogaster* and *E. pyramidum leakeyi* displayed comparable venom complexity, though *E. pyramidum leakeyi* had fewer top proteins. Trypsin digestion consistently produced fewer identifications compared to MELD across all species, validating the added depth MELD provides. As with the *Dendroaspis* venoms, the MELD results were used for in-depth venom characterization, whereas trypsin-based digestion enabled quantitative analysis, maximizing analytical resolution and functional insight.

Following the same classification of proteins, venom proteins were categorized as toxins and non-toxins. As *Echis* and *Dendroaspis* venoms do not contain the same families of toxins, proteins classified as toxins for this analysis were as follows: renin-like aspartic-proteases [34], C-type lectin-like proteins (CTLs), LAAOs, SVMPs, SVSPs, and PLA_2_s. Similarly, the non-toxin category encompasses, as described for *Dendroaspis*, are venom components that, while not directly cytotoxic or enzymatically active in classic envenomation symptoms, contribute to venom spread or the modulation of host responses. For *Echis*, non-toxins encompassed venom endothelial growth factors, snake venom metalloproteinase inhibitors (SVMPi), CRISP, 5′-nucleotidases, nerve growth factors (NGF), and phosphodiesterases.

**Figure 3:**
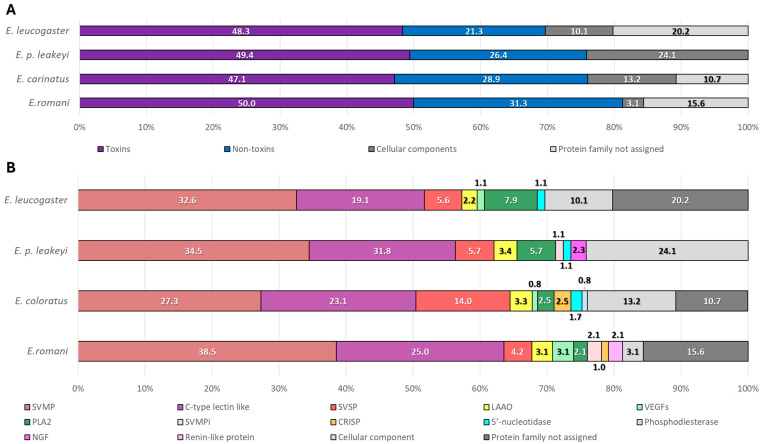
(**A**) Overview of the composition of toxins, non-toxins, cellular components, and unassigned protein families for each *Echis* venom. (**B**) Detailed breakdown of venom component classes for each species, highlighting the most prevalent classes such as SVMPs, C-type lectin-like proteins, and SVSPs.

Figure 3A illustrates the distribution of toxins, non-toxins, cellular components, and protein families not assigned for the four *Echis* snakes. The MELD approach allowed for the identification of a significant proportion of toxins in *Echis* venoms, constituting nearly 50% of all identified proteins. Toxins constituted the largest proportion in all samples, ranging from 47.1% in *Ec* (57 sequences) to 50.0% in *Er* (48 sequences). *Epl* and *El* had similar toxin proportions, at 49.4% (43 sequences) and 48.3% (43 sequences), respectively.

Non-toxins showed considerable variability, with *Er* having the highest proportion (31.3%, 30 sequences) and *El* the lowest (21.3%, 19 sequences). *Ec* and *Epl* fell in between, with 28.9% (35 sequences) and 26.4% (23 sequences), respectively. Cellular components exhibited a wide range, from 3.1% in *Er* (three sequences) to 24.1% in *Epl* (21 sequences), with *Ec* (13.2%, 16 sequences) and *El* (10.1%, nine sequences) having intermediate values. The category of protein families not assigned showed notable differences, being completely absent in *Epl*, but highest in *El* (20.2%, 18 sequences). *Er* and *Ec* had 15.6% (15 sequences) and 10.7% (13 sequences), respectively.

Figure 3B showcases the detailed composition of toxins and non-toxins of the venoms. The major toxin classes identified included SVMPs, SVSPs, CTLs, and LAAOs. SVMPs were most abundant in *Er* (38.5%, 37 sequences) and least in *Ec* (27.3%, 33 sequences), with *Epl* and *El* at 34.5% (30 sequences) and 32.6% (29 sequences), respectively. C-type lectin-like proteins followed, with *Er* having the highest proportion (25.0%, 24 sequences), *El* at 19.1% (17 sequences), and *Ec* and *Epl* at 23.1% (28 sequences) and 21.8% (19 sequences), respectively. These findings align with the protein mass observations from the 1D Gel (Figure 1). SVSP showed significant variability, peaking at 14.0% in *Ec* (17 sequences) and dropping to 4.2% in *Er* (four sequences), with *Epl* and *El* at 5.7% (five sequences) and 5.6% (five sequences), respectively. LAAOs were relatively stable across species, ranging from 2.2% in *El* (two sequences) to 3.4% in *Epl* (three sequences). Venom endothelial growth factors were present in *Er* (3.1%, three sequences) and *El* (1.1%, one sequence), but not detected in *Epl* and nearly absent in *Ec* (0.8%, one sequence).

PLA_2_s were found in every *Echis* venom, peaking at 7.9% in *El* (seven sequences) and reaching a low of 2.1% in *Er* (two sequences), with *Ec* and *Epl* at 2.5% (three sequences) and 5.7% (five sequences), respectively. Certain venom components seem to exhibit species-specific distributions. For example, phosphodiesterases were exclusively found in *Ec* (0.8%, one sequence), renin-like aspartic-proteases in *Er* (2.1%, two sequences), and nerve growth factors solely present in *Epl* (2.3%, two sequences). SVMPis were present in *Er* (2.1%, two sequences) and *Epl* (1.1%, one sequence) but not detected in *Ec* and *El*. Cysteine-rich venom proteins were identified in *Er* (1.0%, one sequence) and *Ec* (2.5%, three sequences), but seem absent in *Epl* and *El*. These findings highlight significant interspecies variations in venom composition, with *Er* and *Ec* exhibiting the most diverse venom profiles. In contrast, *El* and *Epl* displayed moderate complexity in terms of number of sequences identified.

### 2.4. Proteomics Relative Toxin Quantification for Echis and Dendroaspis Venoms

**Figure 4:**
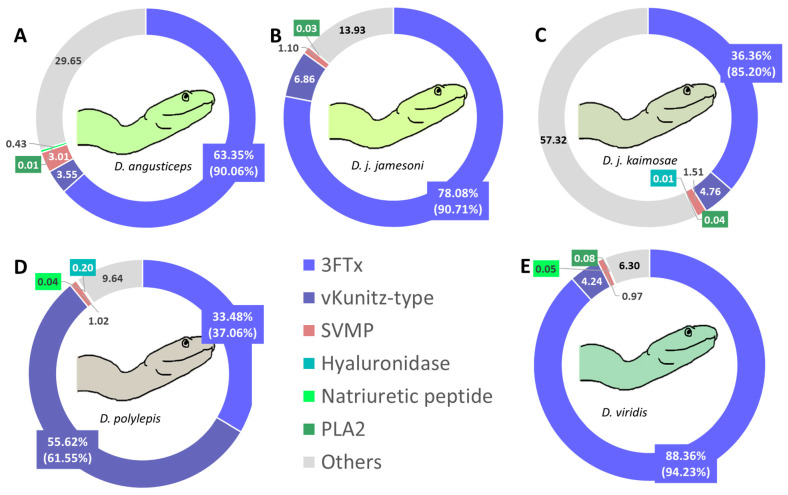
Relative quantification of the most relevant toxins for the five *Dendroaspis* venoms: (**A**) *D. angusticeps*, (**B**) *D. j. jamesoni*, (**C**) *D. j. kaimosae*, (**D**) *D. polylepis*, (**E**) *D. viridis*. Relative quantification was performed by relative proportion area after tryptic digestion and grouped by toxin family identifications. Others refer to non-toxins (including cellular components) and unknown proteins. The values in brackets indicate the recalculated percentages of the most prevalent toxin families when the category “Others” is not considered. Where fractions were too small to be visible in the pie chart, the relevant percentage is indicated adjacent to the fraction.

While the MELD strategy enabled a deeper identification of both abundant and low-abundance toxins, the relative quantification of toxin families was derived from trypsin-only digestion. This quantification was based on the relative area of unique peptides obtained by shotgun proteomics using the LC-MS data acquired (Figures S7–S15), which were assigned to their corresponding parent toxins. This label-free peptide-based method allows for a proportional estimation of each toxin family’s contribution within the venom. Toxin quantification, based on the relative proportion area of unique peptides’ chromatographic peaks from the parent protein, revealed significant variability among all *Dendroaspis* species (Figure 4). 3FTxs were the most abundant components, particularly in *D. viridis* (*Dv*, 88.36%), *D. j. jamesoni* (*Djj*, 78.08%), and *D. angusticeps* (*Da*, 63.35%), while lower proportions were observed in *D. j. kaimosae* (*Djk*, 36.36%) due to a large proportion of the family “others” and *D. polylepis* (*Dp*, 33.48%). vKunitz-type proteins exhibited considerable abundance in *Dp* (55.62%), while other samples showed lower proportions, ranging from 3.55% in Da to 6.86% in *Djj*. SVMPs showed the highest prevalence in *Da* (3.01%) and the lowest in *Dv* (0.98%). Natriuretic peptides were detected in low quantities, with *Da* having 0.43%, *Dv* 0.05%, and *Dp* 0.04%, while these were not detected in the other venoms. The PLA_2_s levels were minimal across all samples, with the highest recorded in Dv (0.08%). Hyaluronidase was only detected in *Dp* (0.20%). The “Others” category, including non-toxin and unknown proteins, varied widely, being most prominent in *Djk* (57.32%) and least in *Dv* (6.30%).

**Figure 5:**
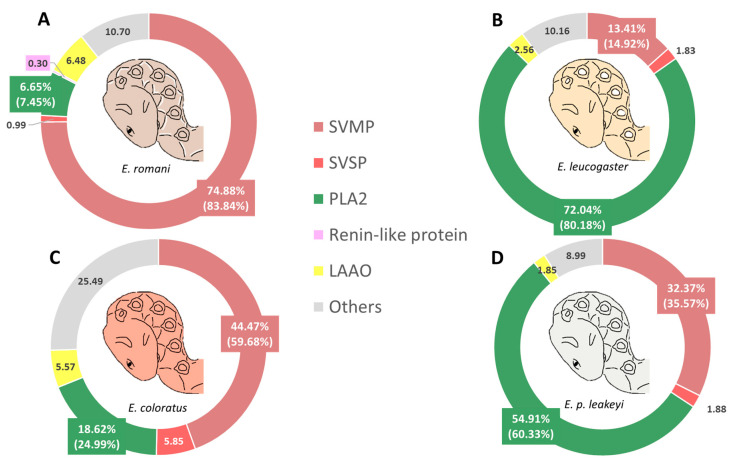
Relative quantification of the most relevant toxins for the four *Echis* venoms: (**A**) *E. romani*, (**B**) *E. leucogaster*, (**C**) *E. coloratus*, (**D**) *E. p. leakeyi*. Relative quantification was performed by relative proportion area after tryptic digestion and grouped by toxin family identifications. Others refer to non-toxins (including cellular components) and unknown proteins. The values in brackets indicate the recalculated percentages of the most prevalent toxin families when the fraction “Others” is not considered. Where fractions were too small to be visible in the pie chart, the relevant percentage is indicated adjacent to the fraction.

In contrast, *Echis* venoms displayed distinct toxin compositions (Figure 5). SVMPs were most abundant in *E. romani* (*Er*, 74.88%), with considerable proportions in *E. coloratus* (*Ec*, 44.47%) and *E. p. leakeyi* (*Epl*, 32.37%), but significantly lower levels in *E. leucogaster* (*El*, 13.41%). PLA_2_s dominated *El* (72.04%) and *Epl* (54.91%), while *Ec* (18.62%) and *Er* (6.65%) showed moderate levels. LAAOs displayed notable variability, being highest in *Er* (6.48%) and *Ec* (5.57%), while lower levels were observed in *El* (2.56%) and *Epl* (1.85%). SVSPs were detected in low proportions, ranging from 0.99% in *Er* to 5.85% in *Ec*. Renin-like aspartic-proteases were exclusively found in *Er* (0.30%) and absent in other samples. The “Others “ category exhibited significant variability, with the highest proportion in *Ec* (25.49%) and the lowest in *Epl* (8.99%), while *Er* and *El* showed similar values of 10.70% and 10.16%, respectively.

## 3. Discussion

Trypsin digestion and MELD serve distinct roles in venom proteomics, with key differences in their application for protein identification and quantification. Trypsin digestion is the standard approach in proteomics due to its high specificity, cleaving proteins at lysine and arginine residues, generating consistent and predictable peptides for MS analysis. This predictability makes trypsin particularly suitable for relative quantification based on the relative proportion area of unique peptides from parent toxins. Since trypsin digestion produces a consistent set of peptides per protein, it enables accurate toxin family quantification by measuring unique peptide intensities corresponding to their parent proteins. However, a paucity of cleavage sites can restrict sequence coverage, leading to incomplete protein identification, especially for venom proteins with structural constraints. MELD digestion, on the other hand, enhances protein identification by using multiple proteases, increasing peptide diversity and sequence coverage. This approach is advantageous for detecting low-abundance venom components and improving overall proteome depth. However, due to the multiple cleavage sites across different proteases, MELD generates a large and variable set of peptides per protein, making it incompatible with traditional relative quantification approaches based on unique peptides from a single enzyme digest. The variability in peptide generation means that peptide intensities cannot be directly correlated with parent protein abundance, preventing accurate relative proportion area-based quantification.

This study utilized MELD combined with parallel tryptic digestion to achieve an in-depth characterization of venom components from nine African venomous snakes that collectively contribute to a substantial number of snakebite fatalities in Africa annually. By integrating advanced proteomic techniques, we achieved unprecedented resolutions in venom analysis, uncovering key toxin families and enabling inter-species comparisons. The MELD approach marked a significant methodological advancement, being considered a next-generation sequencing approach for venomics, enhancing the detection and characterization of venom proteins, including those present in low abundance [31]. By applying MELD to different snake species, we confirmed its reproducibility and robustness in venom analysis, reinforcing its value as a next-generation sequencing approach for venomics. This was particularly evident in the identification of rare components, such as hyaluronidases in *D. j. kaimosae* and renin-like aspartic-proteases in *E. romani*, which underscore the approach’s ability to uncover bioactive molecules with potential therapeutic applications. Trypsin digestion complemented MELD by enabling relative proportion area quantification, providing valuable insights into the abundance of toxins and non-toxins across species. A false discovery rate (FDR) of 1% and the presence of at least one unique peptide and significant peptide per sequence were employed for filtering out inaccurate proteins during database searches. The top proteins, supported by the most unique peptides in the group, were utilized for their classification into toxins, non-toxins, protein families not assigned, and cellular components. Venom complexity varied significantly among the species studied, with *D. viridis* and *E. coloratus* exhibiting the highest venom diversity in protein profiles and toxin classes, while *D. polylepis* and *E. leucogaster* displayed comparatively lower diversity. These differences may reflect ecological and evolutionary constraints, as venom composition is thought to be adaptively shaped by dietary and environmental conditions [35,36,37].

Quantification of key toxin families revealed distinct patterns of specialization, with *Dendroaspis* venoms dominated by neurotoxic 3FTxs and vKunitz-type proteins, and *Echis* venoms dominated by hemotoxic SVMPs. The 3FTxs family is the major toxin type in *D. viridis* (88.39%), *D. j. jamesoni* (78.01%), and *D. angusticeps* (63.37%) venoms, contributing to their potent neurotoxic effects. This high abundance is consistent with the rapid onset of symptoms such as muscle paralysis, respiratory failure, and cardiovascular instability, which are hallmark effects of *Dendroaspis* envenomation. In contrast, lower proportions in *D. polylepis* (33.47%) and *D. j. kaimosae* (36.38%) suggest a broader venom profile. 3FTxs are non-enzymatic peptides that are typically of high abundance in elapid snakes. They are characterized by specific folding of three beta-sheet loops (“fingers”) extending from the central core and by four conserved disulfide bridges. The three loops that project from the core region resemble three outstretched fingers of the hand, hence the name. Despite their structural similarity, they differ widely in their targets, which are numerous: L-type calcium channels, sodium channels, integrin receptors, cell membrane phospholipids, acetylcholinesterase, acetylcholine receptors (nicotinic or muscarinic), adrenergic receptors, and dopaminergic receptors [38,39]. Regarding the high proportion of vKunitz-type proteins in *D. polylepis* (55.61%), this toxin family is clinically significant, as these toxins, also known as dendrotoxins, act as voltage-gated potassium channel blockers, contributing to pronounced neurotoxic effects [40] by inhibiting serine proteases involved in hemostasis [41,42], or by blocking potassium channels [43], or even both [44,45,46]. The lower levels detected in other species (e.g., 3.55% in *D. angusticeps*) demonstrate substantial variation in the relative abundance of this toxin type across this genus of snakes. Although SVMPs are present in low amounts across *Dendroaspis* venoms (highest at 3.01% in *D. angusticeps*), their presence may enhance local tissue damage and facilitate venom diffusion. This could contribute to local pain and swelling, though these effects are typically less prominent compared to vipers. However, a case following a bite by *D. angusticeps* described local envenoming with signs of cytotoxicity and hemostatic disturbances, but no neurotoxicity [47]. In summary, this quantitative analysis aligns with previous studies, confirming the dominance of 3FTxs in green mamba venoms—*D. viridis*, *D. j. jamesoni*, and *D. angusticeps*—and the enrichment of vKunitz-type toxins in *D. polylepis* [48,49,50]. Our findings also support the minimal enzymatic profile of mamba venoms, with SVMPs and PLA_2_s only detected at trace levels.

The venom composition of *Echis* species aligns with the severe systemic and local effects reported in human envenomation cases. SVMPs dominate the venoms of *E. romani* (74.88%) and *E. coloratus* (44.47%), contributing to severe hemorrhage, coagulopathy, and local tissue destruction. SVMPs are typically present in large quantities in viperid venoms, though they are also consistently found in elapid and colubrid venoms. Several biological activities have been attributed to them, including hemorrhage, edema, inflammation, hypotension, and necrosis [51,52]. They act by interfering at different levels of hemostasis. The metalloproteinase domain is responsible for the degradation of matrix proteins, which thus results in the weakening of the endothelial cell attachment, and also for the degradation or activation of proteins involved in hemostasis, such as fibrinogen [52]. The lower SVMPs levels observed in *Echis leucogaster* (13.41%) suggest a reduced hemorrhagic potential, which may contribute to milder systemic effects compared to other *Echis* species. Supporting this, *E. leucogaster* venom has been shown to induce slower clotting times and weaker procoagulant effects in vitro [53], aligning with its relatively low metalloproteinase abundance. Interestingly, ethnobiological reports from traditional healers in West Africa have described envenomation by *E. leucogaster* as causing cardiac distress and respiratory difficulties [54]—symptoms not commonly associated with other *Echis* species. This raises the possibility that unique components or concentrations in *E. leucogaster* venom, beyond SVMPs, may influence envenoming manifestations. However, such ethnographic accounts [54] must be interpreted cautiously. Terminologies such as “cardiac troubles” or “breathing problems” [54] can reflect culturally specific understandings of illness, which may not directly correspond to biomedical pathophysiology. PLA_2_s, abundant in *E. leucogaster* (72.04%) and *E. p. leakeyi* (54.91%), exacerbate envenomation effects by stimulating platelet aggregation and causing anti-coagulation [55,56], contributing to bleeding disorders and thrombosis frequently reported during viper envenomation. Cytotoxicity and hemolysis are also common effects, leading to localized tissue necrosis and, in severe cases, amputations [55,56]. Interestingly, lower PLA_2_s levels in *E. romani* (6.65%) and *E. coloratus* (18.62%) indicate a strong variability in PLA_2_ venom composition among *Echis* species. LAAOs, abundant in *E. romani* (6.48%) and *E. coloratus* (5.57%), generate hydrogen peroxide [57], exacerbating oxidative stress, tissue damage, and necrosis. This contributes to swelling, blistering, and rapid tissue destruction, intensifying envenomation severity. Our quantitative analysis revealed that the SVSP levels also varied among *Echis* species, being highest in *E. coloratus* (5.85%), followed by *E. p. leakeyi* (1.88%), *E. leucogaster* (1.83%), and lowest in *E. romani* (0.99%). Despite their low abundance, SVSPs can disrupt coagulation by mimicking thrombin activity, and these toxins may contribute to the overall hemorrhagic effect of *Echis* venoms. Most proteomic studies on *Echis* venoms to date have focused on *E. ocellatus* [28,58,59] and *E. carinatus* [59,60]. As in the study by Casewell et al. (2014) [59], our analysis includes three overlapping *Echis* species—*E. p. leakeyi*, *E. coloratus*, and *E. ocellatus* (note that this was formerly *E. ocellatus*)—enabling comparative insights into venom composition. Our study complements these efforts by providing relative quantitative proteomics using tryptic digestion and relative proportion area measurement and the addition of *E. leucogaster* species. Overall, our findings align with previous studies, as we observed that SVMPs remain the dominant toxin family in *E. romani* and *E. coloratus*, though our relative quantification indicates a higher SVMP proportion in *E. romani* (74.88%) compared to *E. ocellatus* [59]. Conversely, in *E. coloratus*, our SVMP estimate (44.47%) is somewhat lower than that in a previous report [59]. A key contrast emerges in PLA_2_ abundance: *E. leucogaster* and *E. p. leakeyi* exhibited exceptionally high PLA_2_ levels (72.04% and 54.91%, respectively) compared to previously studied *Echis* species. Furthermore, LAAOs were detected in notable quantities in *E. romani* (6.48%) and *E. coloratus* (5.57%) in our study.

## 4. Conclusions

This study provides a comprehensive analysis of the venom composition of nine African venomous snake species from the *Dendroaspis* and *Echis* genera, revealing substantial interspecific diversity and specialization of their venom components. By employing advanced proteomic methodologies, including MELD and tryptic digestion, we achieved enhanced proteome coverage and deeper molecular resolution compared to those of traditional single-enzyme approaches. This allowed for the identification of both abundant and rare toxin families. The integration of these techniques facilitated the detection of novel bioactive molecules, such as hyaluronidases in *D. j. kaimosae* and renin-like aspartic-proteases in *E. romani*. Quantitative comparisons between species underscored key differences in toxin profiles—such as the predominance of 3FTxs in *Dendroaspis* and SVMPs in *Echis*, which are directly relevant for understanding clinical manifestations of envenomation and improving species-specific antivenom design. This study thus provides further evidence that the MELD strategy enables deep characterization of venom component diversity. The annotated toxin database generated in this work will be made publicly available via the ADDovenom platform (https://www.addovenom.com, accessed on 1 May 2025), supporting future comparative and translational research. Altogether, this study not only refines our understanding of venom diversity in medically important African snakes but also lays the groundwork for future translational research in antivenom development while reinforcing the value of combinatory proteomic strategies for uncovering biologically significant, yet previously underexplored, venom components.

## 5. Materials and Methods

Ethical statement, animal care and venoms’ sampling. Eight of the nine venom samples analyzed in this study were sourced from wild-caught specimens maintained in the UK-Home-Office-regulated herpetarium of the Centre for Snakebite Research and Interventions at the Liverpool School of Tropical Medicine (Pembroke Place, Liverpool, UK). This facility and its snake husbandry protocols are approved and inspected by the UK Home Office and the LSTM Animal Welfare and Ethical Review Boards (establishment license No. X20A6D134). All animals were imported into the UK prior to the implementation of the Nagoya protocol. The *Dendroaspis* specimens were wild-caught as young adults and have been maintained in captivity since 2014. The *Echis* specimens wild-caught as young adults were acquired earlier, around 2006. Diet during captivity consisted primarily of defrosted medium-to-large mice, with occasional supplementation of dead day-old chicks. Venoms were provided as species pools from multiple specimens of five species of mamba, *D. angusticeps* (Tanzania), *D. polylepis* (Tanzania), *D. j. jamesoni* (Cameroon), *D. j. kaimosae* (Uganda), and *D. viridis* (Togo), and three species of saw-scaled viper, *E. coloratus* (Egypt), *E. p. leakeyi* (Kenya), and *E. romani* (Nigeria). The final venom, sourced from multiple specimens of *E. leucogaster* (Mali), originated from the Liverpool School of Tropical Medicine’s historical venom collection, and was previously used as a WHO reference venom. All crude venoms were lyophilized and stored at 4 °C or −20 °C prior to analysis. Venom extraction of venomous snakes is not a regulated procedure in the UK. The LSTM holds a UK Home Office institutional license that covers these activities (No. X20A6D134).

Sodium Dodecyl Sulfate Polyacrylamide Gel Electrophoresis (SDS-PAGE): Each venom sample in solution at 5mg/mL (6 μL–30 μg) was mixed with Laemmli buffer containing 700 mM dithiothreitol (DTT, reducing agent; ThermoFisher Scientific (Dilbeek, Belgium) ultrapure molecular biology grade) at a 1:1 ratio (*v/v*) and heated at 100 °C for 3 min. Subsequently, the proteins were separated using a 4–12% NuPAGE MES gel (Thermo Fisher Scientific, Waltham, MA, USA) run at 200 V for 45 min. A molecular weight marker SeeBlue™ Plus2 Pre-stained Protein Standard (Invitrogen, Grand Island, NY, USA), composed of insulin beta-chain (3 kDa), aprotinin (6 kDa), lysozyme (14 kDa), red myoglobin (17 kDa), carbonic anhydrase (28 kDa), alcohol dehydrogenase (38 kDa), glutamic dehydrogenase (49 kDa), bovine serum albumin (62 kDa), phosphorylase (98 kDa), and myosin (188 kDa), was employed. Post-electrophoresis, the gel was initially dehydrated in a solution of 50% ethanol and 3% phosphoric acid (*v/v*) for 3 h, followed by rehydration in ultrapure water (MilliQ) for 20 min. The proteins were stained overnight with Coomassie blue at a concentration of 360 g/L in an aqueous buffer containing 34% methanol (*v/v*), 3% phosphoric acid (*v/v*), and 17% ammonium sulfate (*v/v*). The gel was destained with three consecutive MilliQ water baths (15 min each). Finally, the gel was stored at 5 °C in 5% acetic acid (*v/v*) and subjected to scanning for analysis.

MALDI-TOF MS analysis of crude venom. Venom samples from the nine snake species were analyzed using Matrix-Assisted Laser Desorption/Ionization Time-of-Flight Mass Spectrometry (MALDI-TOF MS) (RapifleX, Bruker, Bremen, Germany) for intact protein profiling. The samples were diluted in ultrapure water with 0.1% formic acid (FA) for optimal ionization. For each sample, 1 μL of venom solution at a concentration of 5 mg/mL was spotted onto the target plate and mixed at a 1:1 ratio with 2,5-dihydroxybenzoic acid (DHB) matrix at 20mg/mL, prepared in 50% acetonitrile and 0.1% FA. The thin-layer deposition method was used to co-crystallize the matrix and sample. Spectra were acquired in linear positive ion mode. For *Dendroaspis* venoms, mass spectra were acquired in two distinct ranges: 600 Da–10 kDa and 1 kDa–25 kDa. For *Echis* venoms, spectral acquisition covered 1 kDa–25 kDa and 20 kDa–100 kDa. The instrument was calibrated with a protein standard calibration mix (protein calibration standard I, Bruker, Billerica, MA, USA). The baseline was subtracted to eliminate background noise, followed by spectral smoothing to improve signal resolution and peak definition.

Multi-enzymatic limited digestion (overview of venom composition). In triplicate, 10 μg of each of the nine lyophilized venoms was solubilized in 20 μL of 50 mM NH_4_HCO_3_ at pH 7.8. Disulfide bonds were reduced using 2 μL of 30 mM dithiothreitol (DTT; Thermo Scientific, ultrapure molecular biology grade) for 40 min at 56 °C, agitated at 650 rpm. Subsequently, the resulting sulfhydryl functions underwent alkylation for 30 min at room temperature in darkness using 3 μL of 60 mM iodoacetamide (IAA; Sigma-Aldrich, BioUltra grade). A subsequent step involved quenching the alkylation by adding 2 μL of 60 mM DTT for 10 min at room temperature in darkness. The MELD approach was implemented to maximize sequence coverage by combining three proteases with complementary cleavage specificities (trypsin [Pierce Trypsin Protease, MS grade, Thermo Scientific], Glu-C [MS grade, Thermo Scientific], and chymotrypsin [MS grade, Thermo Scientific]). Following this, the MELD approach was performed as follows: enzyme solutions were prepared immediately prior to use by combining pure 1 mg/mL solutions at a ratio of 1.00/1.00/1.55 (*v/v*) for trypsin/Glu-C/chymotrypsin. For the high-ratio MELD, the enzyme mixture was directly utilized, while for the low-ratio MELD, it was obtained by diluting the former nine-fold using 25 mM NH_4_HCO_3_ and 5 mM CaCl_2_. Simultaneous digestion was conducted by adding equal volumes of the prepared enzyme mixtures to two separate protein fractions of 10 μg, resulting in final protease-to-protein ratios of 1/85, 1/85, and 1/55 for high-ratio digestion, and 1/750, 1/750, and 1/500 for low-ratio digestion by trypsin, Glu-C, and chymotrypsin, respectively. Each reaction tube was then incubated for 2 h at 37 °C with agitation at 650 rpm using a Thermomixer Comfort (Eppendorf, Hamburg, Germany). The reactions were terminated by the addition of 10% trifluoroacetic acid (TFA) to reach a final pH of approximately 3.0. The digested samples were dried in a speed vacuum concentrator and 3.5 μg was resuspended in 20 μL of 0.1% TFA for desalting using ZipTip pipette tips filled with C_18_ resin. The peptides were eluted with 20 μL of 0.1% TFA/ACN (50/50). The resulting eluates were dried under a speed vacuum and reconstituted to 1.5 μg/9 μL in H_2_O with 0.1% TFA for mass spectrometry injection. Equal volumes of the high- and low-ratio digests were combined to maximize peptide diversity, and the final mixture was analyzed by LC-MS/MS. The use of MELD enables the generation of overlapping peptides from multiple enzymatic cleavages, facilitating deep proteome coverage and improved identification of low-abundance venom components.

Tryptic digestion (relative quantification). As already explained for the MELD sample preparation, 10 μg of each of the nine lyophilized venoms was solubilized in triplicate in 20 μL of 50 mM NH_4_HCO_3_ at pH 7.8. The samples were then reduced with 2 μL of 30 mM dithiothreitol (DTT; Thermo Scientific, ultrapure molecular biology grade) for 40 min at 56 °C with agitation at 650 rpm. Subsequently, the reduced samples underwent alkylation for 30 min at room temperature in the dark using 3 μL of 60 mM iodoacetamide (IAA; Sigma-Aldrich, BioUltra grade). A subsequent step involved quenching the alkylation by adding 2 μL of 60 mM DTT for 10 min at room temperature in darkness. Then, each sample was submitted to two consecutive trypsin digestion steps using Pierce Trypsin Protease, MS grade (Thermo Scientific). The first step involved digestion at a trypsin-to-protein ratio of 1:50, with overnight incubation at 37 °C and shaking at 650 rpm. The following day, a second digestion step was performed with a trypsin-to-protein ratio of 1:100, and acetonitrile was added to a final concentration of 80% (*v/v*) to favor protein unfolding and improve the digestion yield. This sample was incubated at 37 °C for 3 h. The reactions were quenched by acidification with 10% TFA (*v/v*) to reach pH 3.0. The digested samples were dried in a speed vacuum and 3.5 μg was resuspended in 20 μL of 0.1% TFA for desalting using ZipTip pipette tips filled with C_18_ resin. The peptides were eluted with 20 μL of 0.1% TFA/ACN (50/50). The resulting eluates were dried under a speed vacuum and reconstituted to 1 μg/9 μL in H_2_O with 0.1% TFA for mass spectrometry analysis.

Liquid chromatography–mass spectrometry analysis. LC-MS/MS analyses were conducted with an Acquity M-Class UPLC system (Waters) coupled with a Q-Exactive mass spectrometer (Thermo Scientific, Waltham, MA, USA), operating in nano-electrospray positive mode. The trap column employed was a Symmetry C18 5 μm (180 μm × 20 mm), while the analytical column utilized was an HSS T3 C18 1.8 μm (75 μm × 250 mm) (Waters, Corp., Milford, CT, USA). The samples were loaded to the trap column at a rate of 20 μL/min in 98% solvent A (0.1% formic acid in water) for 3 min, followed by separation on the analytical column at a flow rate of 600 nL/min. The separation gradient employed was as follows: initial conditions of 98% A; 5 min at 93% A; 60 min at 70% A; 70 min at 60% A; and maintenance of 15% A for 5 min, after which the column was reconditioned to initial conditions. Solvent B consisted of 0.1% formic acid in acetonitrile, and the total run time was 100 min. The mass spectrometer was operated using a TopN-MSMS method with N set to 12, wherein one Full MS spectrum was acquired, followed by selection of the 12 most intense peaks (excluding singly charged and unassigned charge precursors) from this spectrum for subsequent acquisition of Full MS^2^ spectra. Parameters for MS spectrum acquisition included a mass range from 400 to 1750 *m*/*z*, a resolution of 70,000 (at *m*/*z* 200), and an AGC target of 1 × 10^6^ or maximum injection time of 200 ms. For MS^2^ spectrum acquisition, the parameters included an isolation window of 2.0 *m*/*z*, Normalized Collision Energy (NCE) of 25, a resolution of 17,500, and an AGC target of 1 × 10^5^ or maximum injection time of 50 ms. Key tune parameters for the Q-Exactive mass spectrometer were as follows: a spray voltage of 2.3 kV, capillary temperature set to 270 °C, and S-Lens RF level maintained at 50.0.

Data analysis. Proteins were identified through automated de novo sequencing performed by the proteomics dedicated software Peaks Studio X+ (v10.5, Bioinformatics Solutions InC., Waterloo, Canada) and matched against a protein database constructed from the transcriptomes of each of the eight species available. Venom gland transcriptomes utilized in this study (for all species except for *E. leucogaster*) were previously described (*Echis* [61] and *Dendroaspis* [48]). As no venom gland transcriptome was available for *E. leucogaster*, the transcriptome of the most closely related available species, *E. p. leakeyi*, was used [62]. Carbamidomethylation was designated as a fixed modification, while oxidation (M) was considered a variable modification. An acceptance of up to three missed cleavages was applied for each protein. Tolerances for parent (MS) and fragment (MS/MS) mass errors were set at 5 ppm and 0.015 Da, respectively. To ensure robustness, a false discovery rate (FDR) of 1% was applied, with a criterion of at least one unique peptide and significant peptides to filter out spurious proteins during the PEAKS search algorithms and “*de novo* only” analysis. The matches required a threshold of −10lgP > 20 for confident database matches, and a protein score of 20 or higher was deemed suitable. The score was calculated as −10 times the common logarithm of the *p*-value. Unique peptides, representing high-confidence peptides specific to particular protein groups and absent in other protein groups, were pivotal for reliable identification. At least one unique peptide per protein group was imperative to ensure confidence in the results. Priority was accorded to top-ranked proteins supported by the highest number of unique peptides within their respective groups. To classify the proteins identified in this study according to their families, a BLASTp protocol was employed. Protein sequences from the transcriptome database identified from the proteomics workflow were exported in FASTA format and subjected to sequence alignment against a Uniprot database of known “Snake venom” protein families. The identified proteins were categorized into major toxins (with established functions in envenomation), minor toxins (potentially bioactive molecules with undefined roles in envenomation), cellular components, and protein families lacking assignment to known functions within the database. Venom component classes, including major toxins and minor toxins, were organized based on their respective venom families for comprehensive analysis. The percentage of the venom components in each digested venom was calculated using the following formula [63]:

[*number of proteins* (*protein family*) ÷ *total proteins detected using LC* − *MS*/*MS*] × 100 (1)

## Figures and Tables

**Table 1:** Database search and de novo sequencing for mamba venoms proteome using the venom gland transcriptome database of each species under MELD and tryptic digestion protocols.

Species	*D. angusticeps*		*D. j. jamesoni*		*D. j. kaimosae*		*D. polylepis*		*D. viridis*	
Protocol	*Trypsin*	*MELD*	*Trypsin*	*MELD*	*Trypsin*	*MELD*	*Trypsin*	*MELD*	*Trypsin*	*MELD*
Peptide Spectrum Matches	3395	5513	3684	7315	2837	4757	3728	2932	3311	5589
Peptides	622	1666	581	1940	513	1240	646	951	616	1701
All proteins	122	137	123	158	96	101	121	122	157	187
Top proteins	82	90	90	110	81	82	92	95	112	136

**Table 2:** Database search and de novo sequenced for *Echis* venoms proteome using the transcriptome database under MELD and tryptic digestion protocols.

Species	*E. coloratus*		*E. leucogaster*		*E. p. leakeyi*		*E. romani*	
Protocol	*Trypsin*	*MELD*	*Trypsin*	*MELD*	*Trypsin*	*MELD*	*Trypsin*	*MELD*
Peptide Spectrum Matches	7418	14632	5180	9461	4943	9364	6860	10377
Peptides	1715	4126	1033	2584	1118	3007	1536	3104
All proteins	130	144	112	140	115	136	108	124
Top proteins	98	121	65	89	74	87	87	96

## Data Availability

The original contributions presented in this study are included in the article/Supplementary Material. Further inquiries can be directed to the corresponding author.

## Supplementary Materials

The following supporting information can be downloaded at: https://www.mdpi.com/article/10.3390/toxins17050243/s1, Figure S1: Cladograms representing the evolutionary relationships within the genera *Dendroaspis* (mambas) and *Echis* (saw-scaled vipers), inspired from Ashraf M. R. et al. (https://dx.doi.org/10.17582/journal.pjz/20190710090746) and Ainsworth S. et al. (https://doi.org/10.1016/j.jprot.2017.08.016), respectively. Figure S2: Uncropped SDS-PAGE of the nine snake venoms studied. The SDS-PAGE gel displays protein profiles from nine venom samples (30 μg each) belonging to the genera *Dendroaspis* (left, five species) and *Echis* (right, four species), alongside molecular weight markers (MM) for reference (6 μg). Protein molecular weights range from 6 kDa to 188 kDa, allowing for the identification of protein bands corresponding to key venom components. Lanes *Da* and *Djj*: venoms from *D. angusticeps* and *D. j. jamesoni* respectively. Lanes *Djk* and *p*: venoms from *D. j. kaimosae* and *D. polylepis*. Lane *Dv*: venom from *D. viridis.* Lanes *Er* and *Ec*: venoms from *E. romani* and *E. coloratus*. Lane *Epl* and *El*: venoms from *E. p. leakeyi* and *E. leucogaster*. Figure S3: Uncropped SDS-PAGE of the five *Dendroaspis* venoms studied. The SDS-PAGE gel displays protein profiles from five venom samples (30 μg each) belonging to the genera *Dendroaspis*. Molecular weight markers lane (MM) for reference (6 μg). Protein molecular weights range from 6 kDa to 188 kDa, allowing for the identification of protein bands corresponding to key venom components. Lanes *Dp* and *Dv*: venoms from *D. polylepis* and *D. viridis* respectively. Lanes *Djj* and *Djk*: venoms from *D. j. jamesoni* and *D. j. kaimosae.* Lane *Da*: venom from *D. angusticeps.* Lane annotated X are empty. Figure S4: Uncropped SDS-PAGE of the four Echis venoms studied. The SDS-PAGE gel displays protein profiles from four venom samples (30 μg each) belonging to the genera *Echis*. Molecular weight markers lane (MM) for reference (6 μg). Protein molecular weights range from 6 kDa to 188 kDa, allowing for the identification of protein bands corresponding to key venom components. Lanes *Er* and *Ec*: venoms from *E. romani* and *E. coloratus*. Lane *Epl* and *El*: venoms from *E. p. leakeyi* and *E. leucogaster*. Lane annotated X are empty. Figure S5: MALDI-TOF MS analysis of *Dendroaspis* venoms: mass spectra of crude venoms from *D. angusticeps*, *D. j. jamesoni*, *D. j. kaimosae*, *D. polylepis* and *D. viridis*. Mass spectra were acquired in two distinct mass ranges: (A) 600 Da–10 kDa and (B) 1 kDa–25 kDa. Spectra were recorded in linear positive ion mode, and peaks correspond to intact proteins and peptides present in the venom. Figure S6: MALDI-TOF MS analysis of *Echis* venoms: Representative mass spectra of crude venoms from *E. coloratus*, *E. leucogaster*, *E. romani* and *E. p. leakeyi*. Mass spectra were acquired in two mass ranges: (A) 1 kDa–25 kDa and (B) 20 kDa–100 kDa. Spectra were recorded in linear positive ion mode, revealing the venom protein composition of each species. Figure S7: Peptide distribution plots from LC-MS analyses of *D. angusticeps* venom samples. (A) Peptide mapping following trypsin-only digestion, in triplicate. (B) Peptide mapping obtained via MELD strategy, in triplicate. Each blue dot represents a peptide identified. The x-axis denotes the peptide mass-to-charge ratio (*m*/*z*), while the y-axis indicates the retention time (in minutes) during chromatographic separation. The unique peptides were used for relative quantification of toxin families in the tryptic digestion dataset. Figure S8: Peptide distribution plots from LC-MS analyses of *D. j. jamesoni* venom samples. (A) Peptide mapping following trypsin-only digestion, in triplicate. (B) Peptide mapping obtained via MELD strategy, in triplicate. Each blue dot represents a peptide identified. The x-axis denotes the peptide mass-to-charge ratio (*m*/*z*), while the y-axis indicates the retention time (in minutes) during chromatographic separation. The unique peptides were used for relative quantification of toxin families in the tryptic digestion dataset. Figure S9: Peptide distribution plots from LC-MS analyses of *D. j. kaimosae* venom samples. (A) Peptide mapping following trypsin-only digestion, in triplicate. (B) Peptide mapping obtained via MELD strategy, in triplicate. Each blue dot represents a peptide identified. The x-axis denotes the peptide mass-to-charge ratio (*m*/*z*), while the y-axis indicates the retention time (in minutes) during chromatographic separation. The unique peptides were used for relative quantification of toxin families in the tryptic digestion dataset. Figure S10: Peptide distribution plots from LC-MS analyses of *D. polylepis* venom samples. (A) Peptide mapping following trypsin-only digestion, in triplicate. (B) Peptide mapping obtained via MELD strategy, in triplicate. Each blue dot represents a peptide identified. The x-axis denotes the peptide mass-to-charge ratio (*m*/*z*), while the y-axis indicates the retention time (in minutes) during chromatographic separation. The unique peptides were used for relative quantification of toxin families in the tryptic digestion dataset. Figure S11: Peptide distribution plots from LC-MS analyses of *D. viridis* venom samples. (A) Peptide mapping following trypsin-only digestion, in triplicate. (B) Peptide mapping obtained via MELD strategy, in triplicate. Each blue dot represents a peptide identified. The x-axis denotes the peptide mass-to-charge ratio (*m*/*z*), while the y-axis indicates the retention time (in minutes) during chromatographic separation. The unique peptides were used for relative quantification of toxin families in the tryptic digestion dataset. Figure S12: Peptide distribution plots from LC-MS analyses of *E. coloratus* venom samples. (A) Peptide mapping following trypsin-only digestion, in triplicate. (B) Peptide mapping obtained via MELD strategy, in triplicate. Each blue dot represents a peptide identified. The x-axis denotes the peptide mass-to-charge ratio (*m*/*z*), while the y-axis indicates the retention time (in minutes) during chromatographic separation. The unique peptides were used for relative quantification of toxin families in the tryptic digestion dataset. Figure S13: title Peptide distribution plots from LC-MS analyses of *E. leucogaster* venom samples. (A) Peptide mapping following trypsin-only digestion, in triplicate. (B) Peptide mapping obtained via MELD strategy, in triplicate. Each blue dot represents a peptide identified. The x-axis denotes the peptide mass-to-charge ratio (*m*/*z*), while the y-axis indicates the retention time (in minutes) during chromatographic separation. The unique peptides were used for relative quantification of toxin families in the tryptic digestion dataset. Figure S14: Peptide distribution plots from LC-MS analyses of *E. romani* venom samples. (A) Peptide mapping following trypsin-only digestion, in triplicate. (B) Peptide mapping obtained via MELD strategy, in triplicate. Each blue dot represents a peptide identified. The x-axis denotes the peptide mass-to-charge ratio (*m*/*z*), while the y-axis indicates the retention time (in minutes) during chromatographic separation. The unique peptides were used for relative quantification of toxin families in the tryptic digestion dataset. Figure S15: Peptide distribution plots from LC-MS analyses of *E. p. leakeyi* venom samples. (A) Peptide mapping following trypsin-only digestion, in triplicate. (B) Peptide mapping obtained via MELD strategy, in triplicate. Each blue dot represents a peptide identified. The x-axis denotes the peptide mass-to-charge ratio (*m*/*z*), while the y-axis indicates the retention time (in minutes) during chromatographic separation. The unique peptides were used for relative quantification of toxin families in the tryptic digestion dataset.
