# Supplementary material for: Dual Proteomics Strategies to Dissect and Quantify the Components of Nine Medically Important African Snake Venoms
Source: Toxins (Basel). 2025 May 13;17(5):243. doi: 10.3390/toxins17050243 (PMC12116074; doi:10.3390/toxins17050243)
Supplement: Supplementary file 1 [file toxins-17-00243-s001.zip › VF-toxins-3590381-SupMat.pdf]

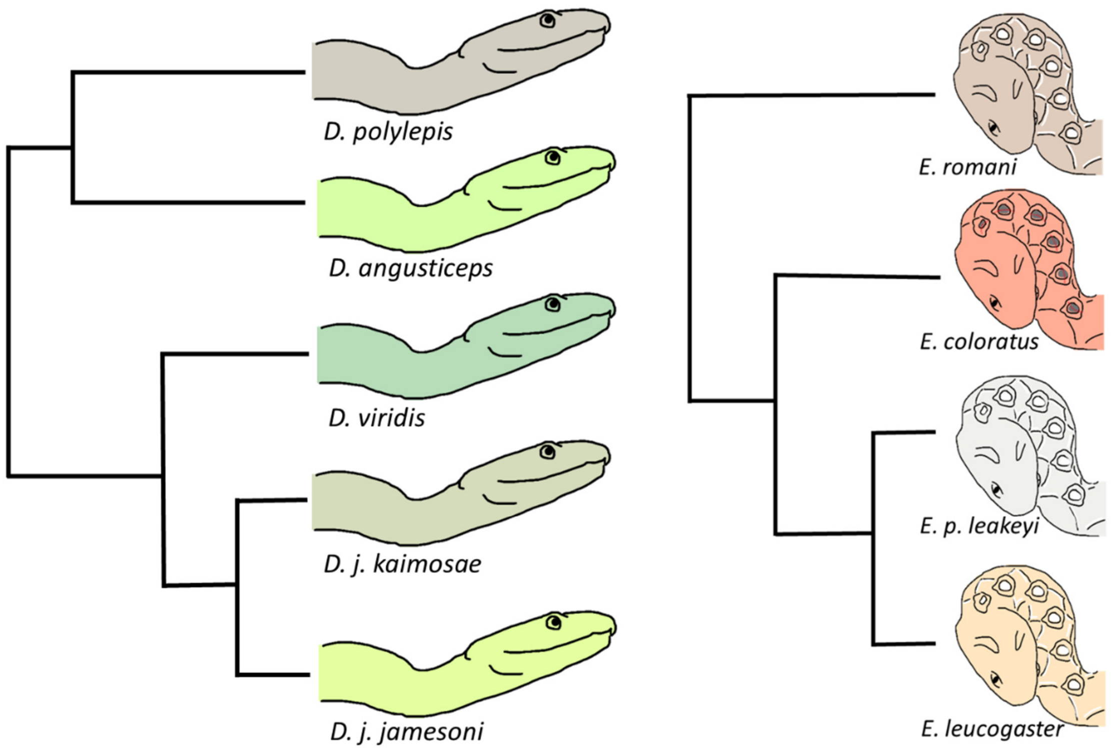

Figure S1. Cladograms representing the evolutionary relationships within the genera *Dendroaspis* (mambas) and *Echis* (saw-scaled vipers), inspired from Ashraf M. R.et al (DOI: https://dx.doi.org/10.17582/journal.pjz/20190710090746) and Ainsworth S. et al. (DOI: https://doi.org/10.1016/j.jprot.2017.08.016), respectively.

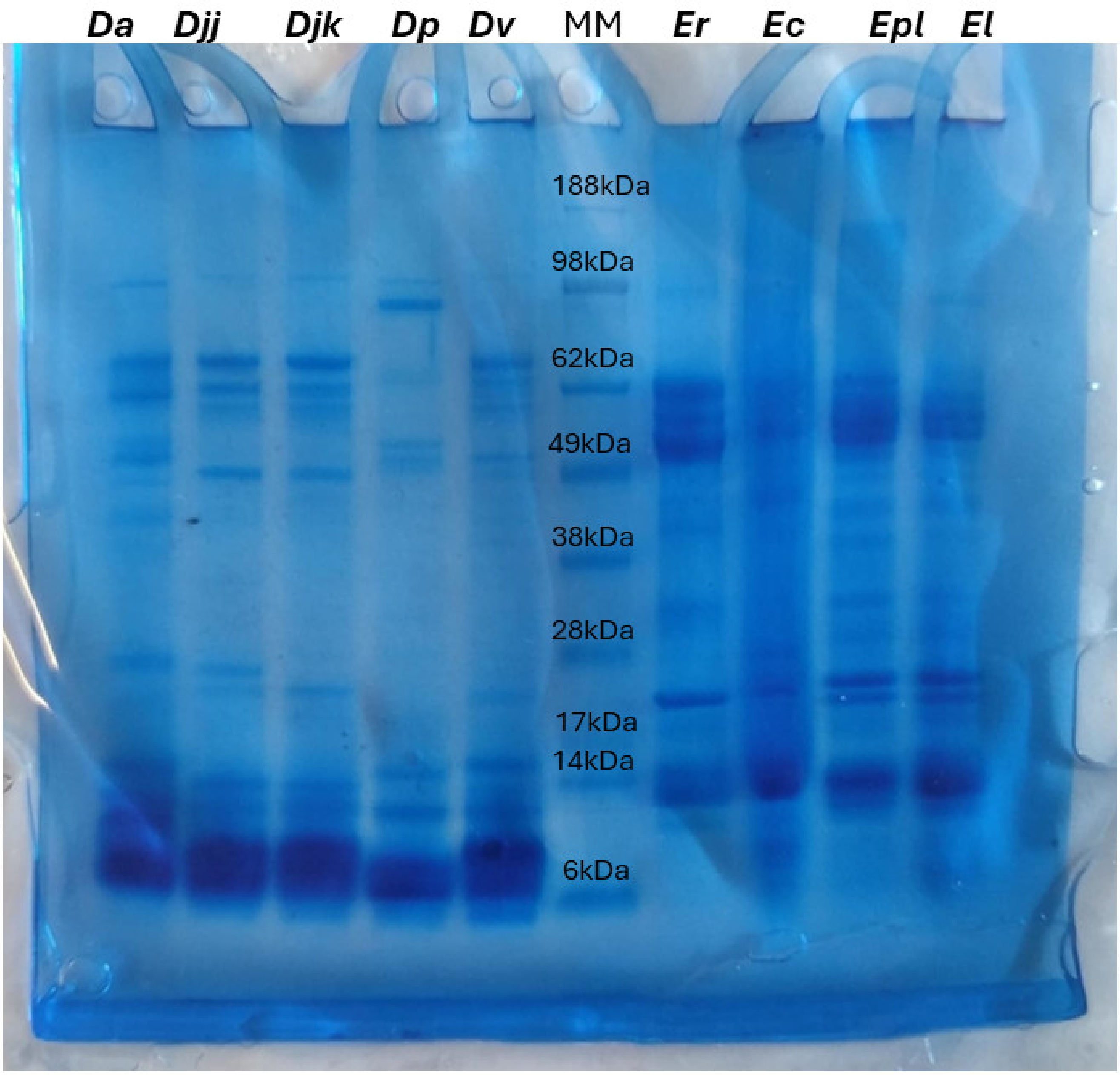

Figure S2. Uncropped SDS-PAGE of the nine snake venoms studied. The SDS-PAGE gel displays protein profiles from nine venom samples (30µg each) belonging to the genera *Dendroaspis* (left, five species) and *Echis* (right, four species), alongside molecular weight markers (MM) for reference (6µg). Protein molecular weights range from 6 kDa to 188 kDa, allowing for the identification of protein bands corresponding to key venom components. Lanes *Da* and *Djj*: venoms from *D. angusticeps* and *D. j. jamesoni* respectively. Lanes *Djk* and *p*: venoms from *D. j. kaimosae* and *D. polylepis*. Lane *Dv*: venom from *D. viridis.* Lanes *Er* and *Ec*: venoms from *E. romani* and *E. coloratus*. Lane *Epl* and *El*: venoms from *E. p. leakeyi* and *E. leucogaster*.

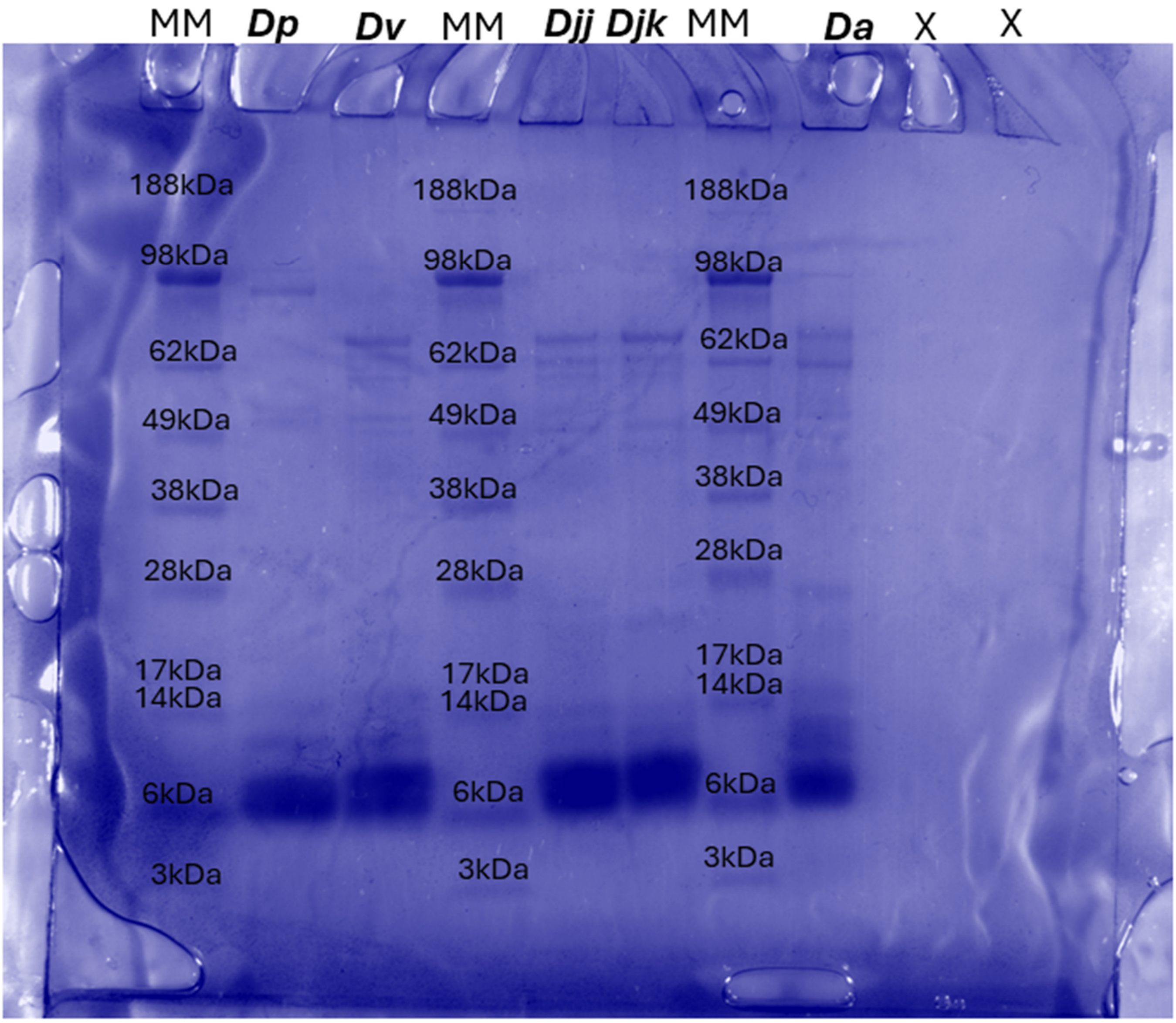

Figure S3. Uncropped SDS-PAGE of the five *Dendroaspis* venoms studied. The SDS-PAGE gel displays protein profiles from five venom samples (30µg each) belonging to the genera *Dendroaspis*. Molecular weight markers lane (MM) for reference (6µg). Protein molecular weights range from 6 kDa to 188 kDa, allowing for the identification of protein bands corresponding to key venom components. Lanes *Dp* and *Dv*: venoms from *D. polylepis* and *D. viridis* respectively. Lanes *Djj* and *Djk*: venoms from *D. j. jamesoni* and *D. j. kaimosae.* Lane *Da*: venom from *D. angusticeps.* Lane annotated X are empty.

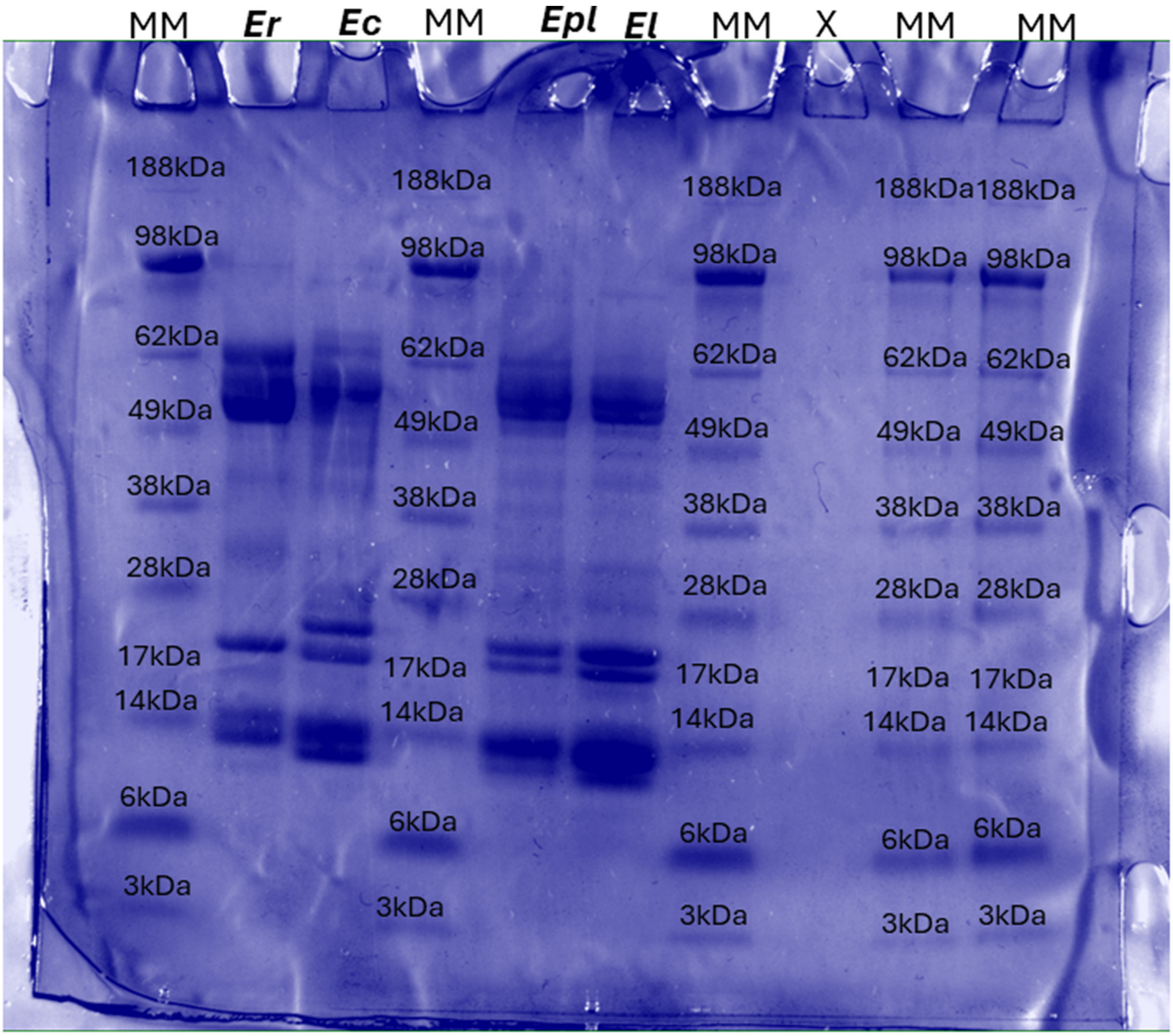

Figure S4. Uncropped SDS-PAGE of the four Echis venoms studied. The SDS-PAGE gel displays protein profiles from four venom samples (30µg each) belonging to the genera *Echis*. Molecular weight markers lane (MM) for reference (6µg). Protein molecular weights range from 6 kDa to 188 kDa, allowing for the identification of protein bands corresponding to key venom components. Lanes *Er* and *Ec*: venoms from *E. romani* and *E. coloratus*. Lane *Epl* and *El*: venoms from *E. p. leakeyi* and *E. leucogaster*. Lane annotated X are empty.

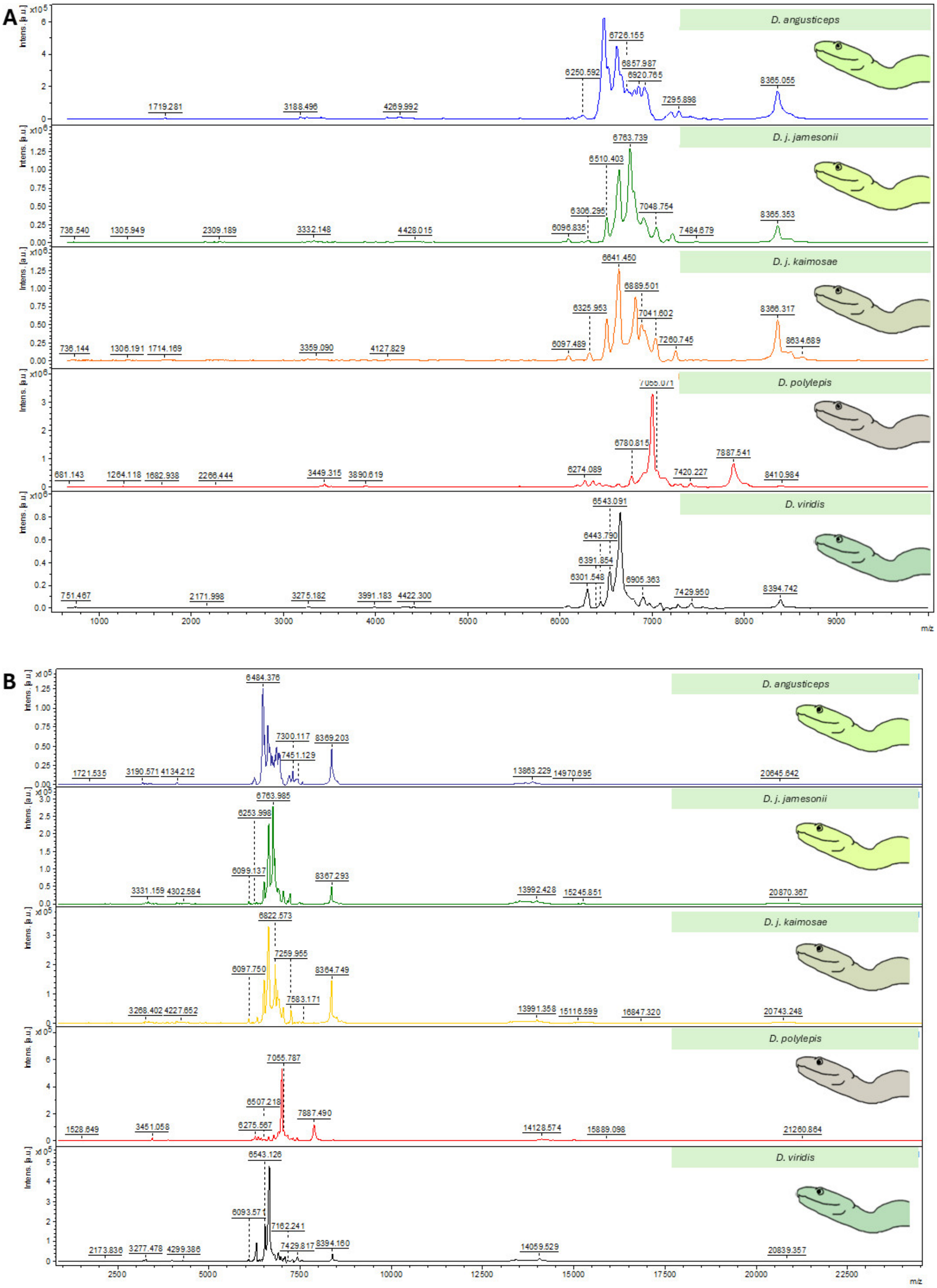

Figure S5: MALDI-TOF MS analysis of *Dendroaspis* venoms: mass spectra of crude venoms from *D. angusticeps*, *D. j. jamesoni*, *D. j. kaimosae*, *D. polylepis* and *D. viridis*. Mass spectra were acquired in two distinct mass ranges: (A) 600 Da – 10 kDa and (B) 1 kDa – 25 kDa. Spectra were recorded in linear positive ion mode, and peaks correspond to intact proteins and peptides present in the venom.

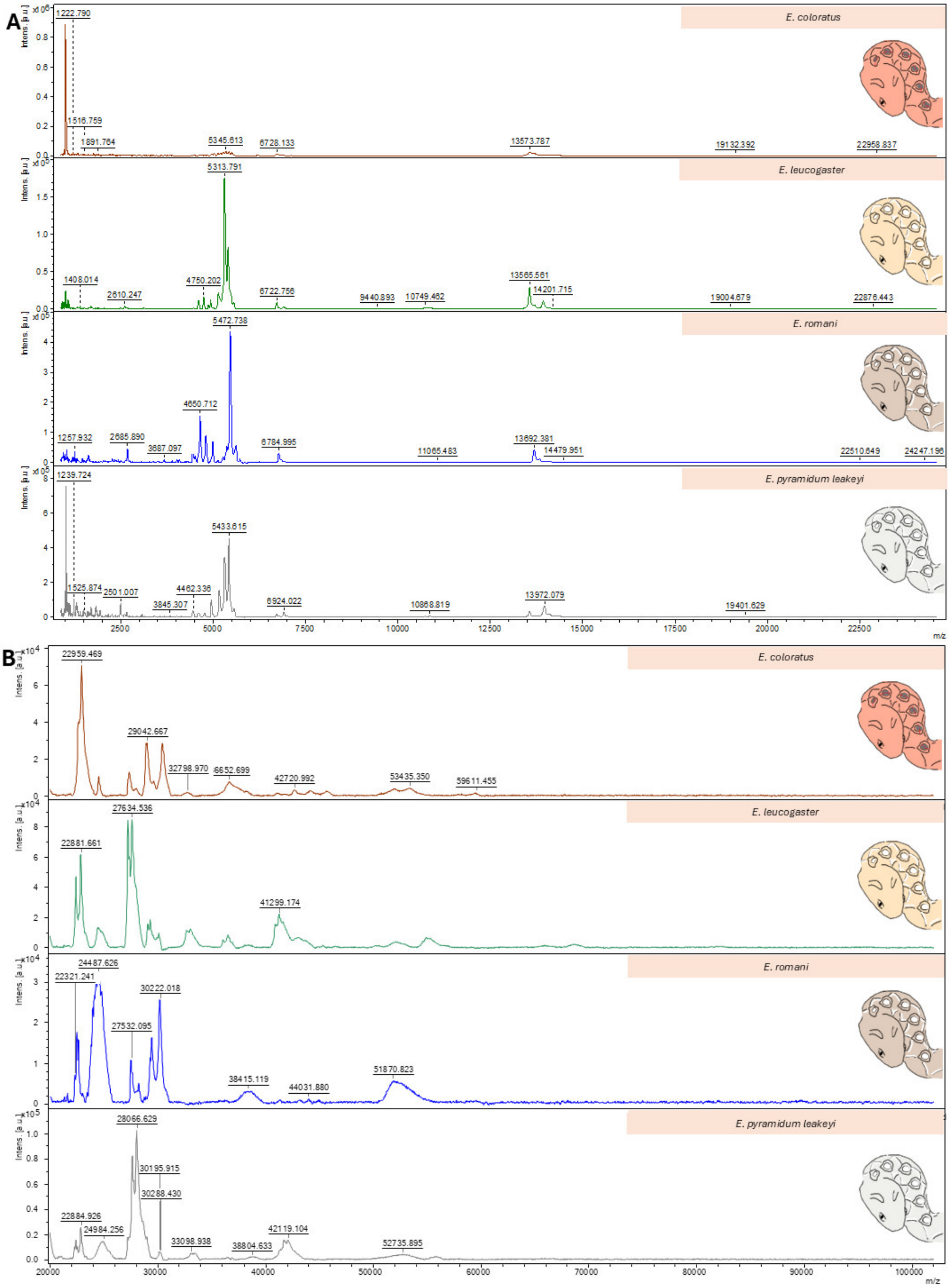

Figure S6: MALDI-TOF MS analysis of *Echis* venoms: Representative mass spectra of crude venoms from *E. coloratus*, *E. leucogaster*, *E. romani* and *E. p. leakeyi*. Mass spectra were acquired in two mass ranges: (A) 1 kDa – 25 kDa and (B) 20 kDa – 100 kDa. Spectra were recorded in linear positive ion mode, revealing the venom protein composition of each species.

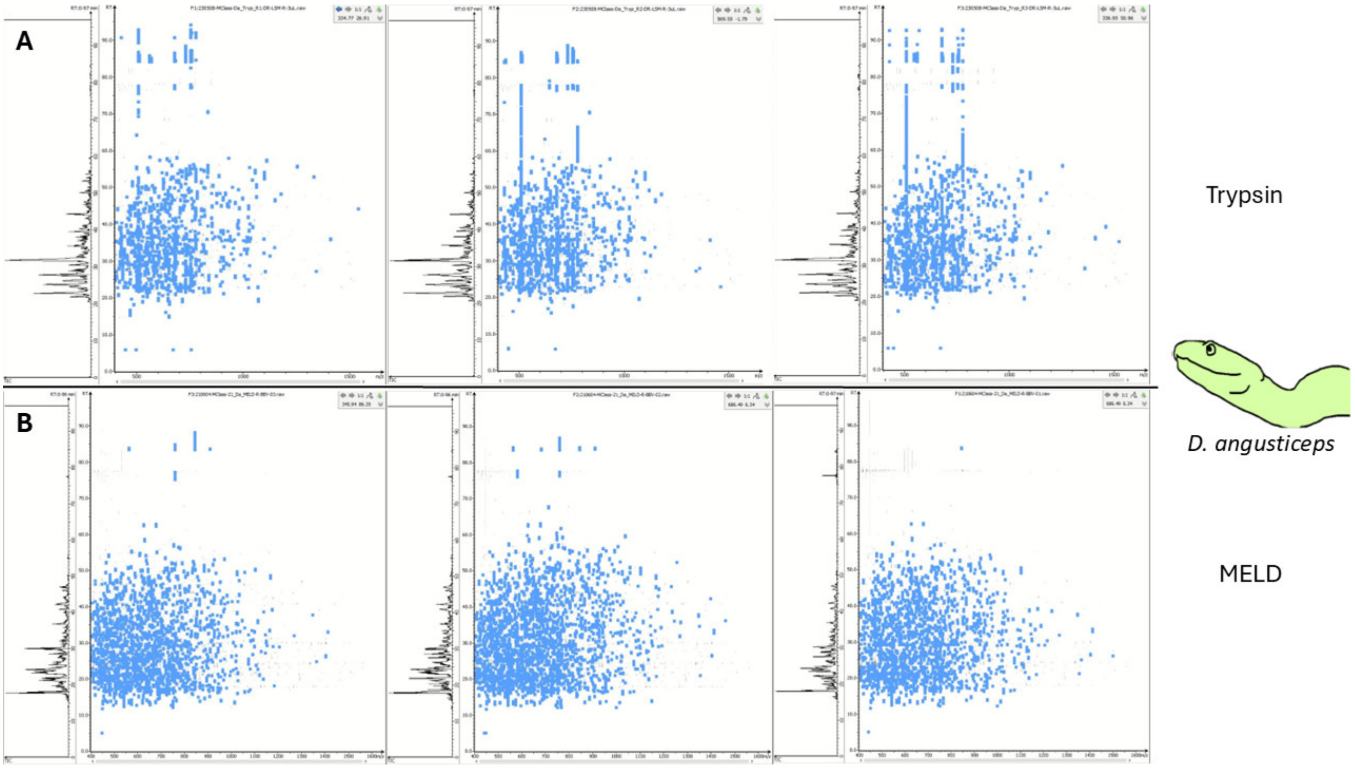

Figure S7. Peptide distribution plots from LC-MS analyses of *D. angusticeps* venom samples. **(A)** Peptide mapping following trypsin-only digestion, in triplicate. **(B)** Peptide mapping obtained via MELD strategy, in triplicate. Each blue dot represents a peptide identified. The x-axis denotes the peptide mass-to-charge ratio (*m/z*), while the y-axis indicates the retention time (in minutes) during chromatographic separation. The unique peptides were used for relative quantification of toxin families in the tryptic digestion dataset.

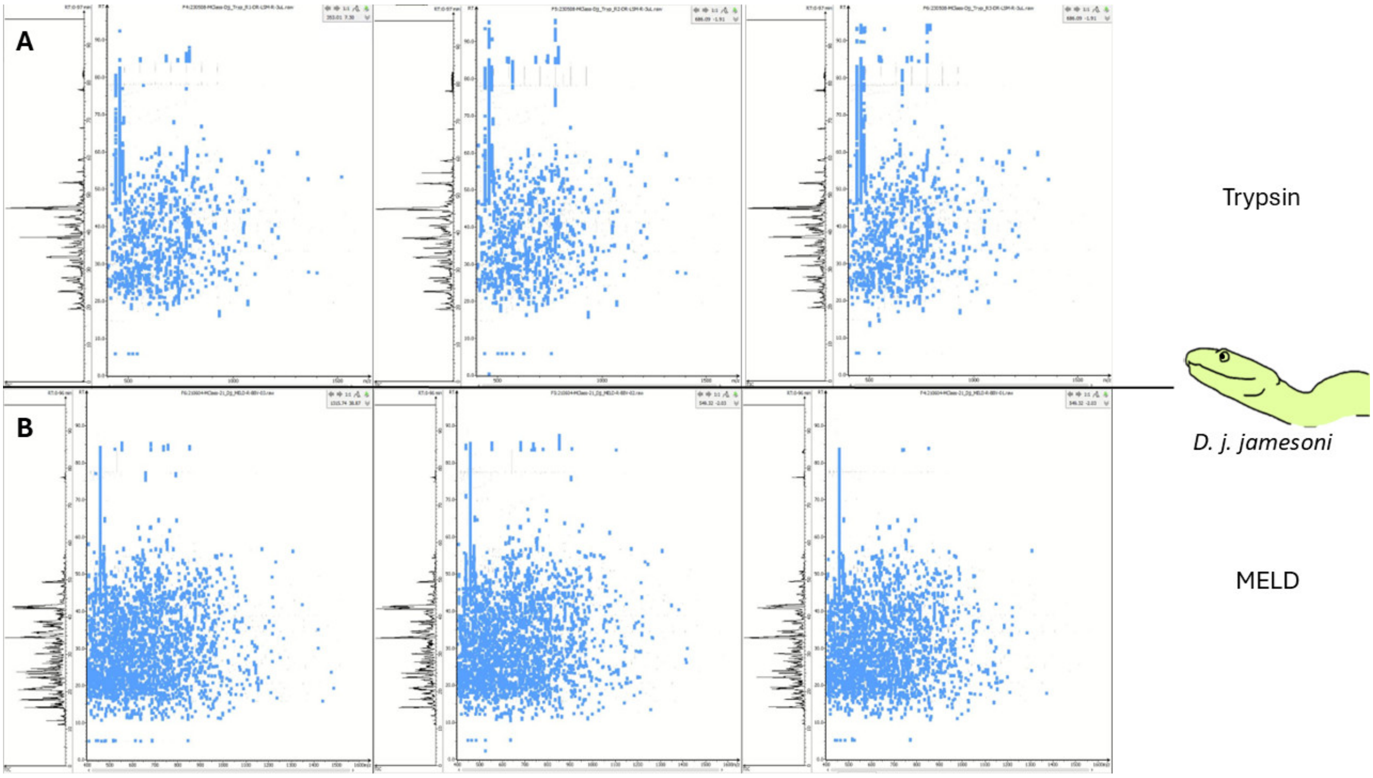

Figure S8. Peptide distribution plots from LC-MS analyses of *D. j. jamesoni* venom samples. **(A)** Peptide mapping following trypsin-only digestion, in triplicate. **(B)** Peptide mapping obtained via MELD strategy, in triplicate. Each blue dot represents a peptide identified. The x-axis denotes the peptide mass-to-charge ratio (*m/z*), while the y-axis indicates the retention time (in minutes) during chromatographic separation. The unique peptides were used for relative quantification of toxin families in the tryptic digestion dataset.

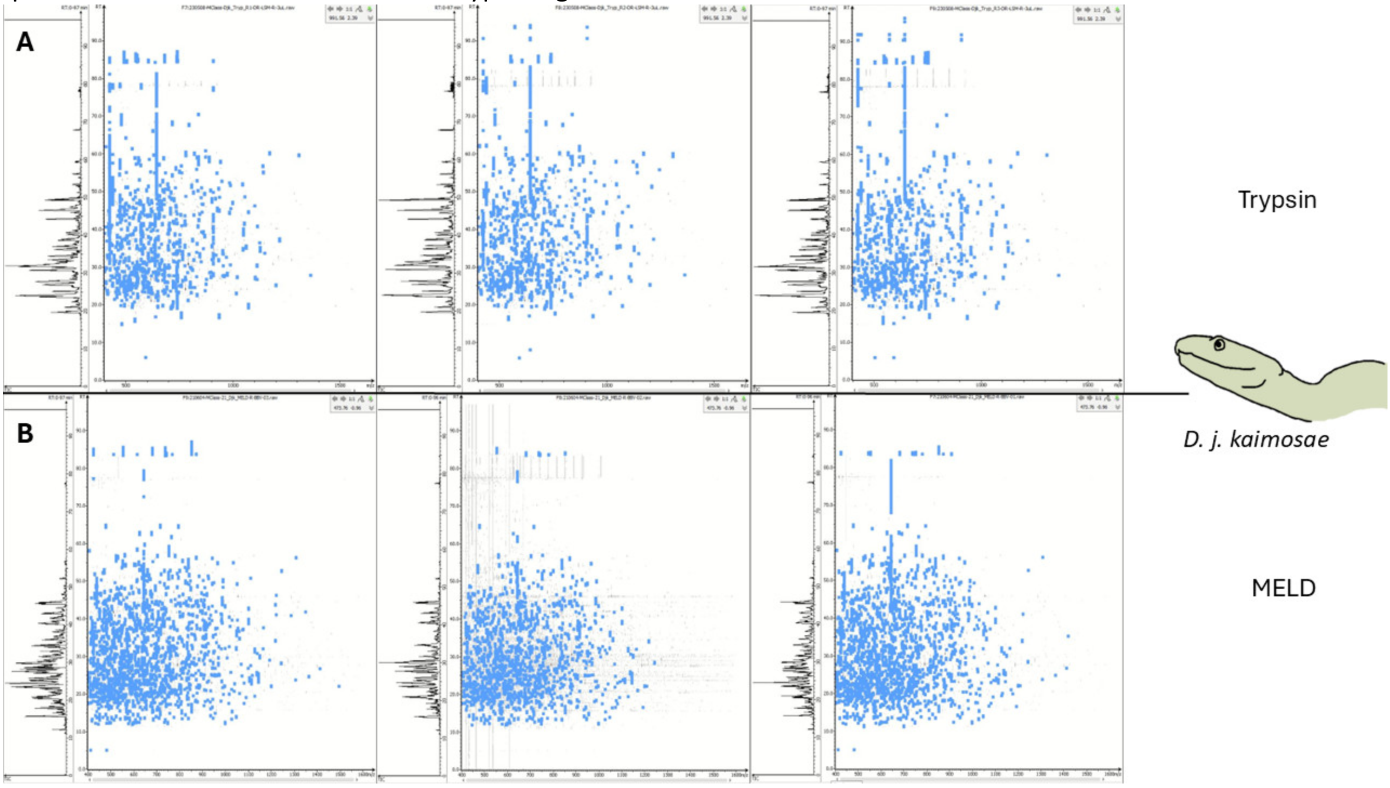

Figure S9. Peptide distribution plots from LC-MS analyses of *D. j. kaimosae* venom samples. **(A)** Peptide mapping following trypsin-only digestion, in triplicate. **(B)** Peptide mapping obtained via MELD strategy, in triplicate. Each blue dot represents a peptide identified. The x-axis denotes the peptide mass-to-charge ratio (*m/z*), while the y-axis indicates the retention time (in minutes) during chromatographic separation. The unique peptides were used for relative quantification of toxin families in the tryptic digestion dataset.

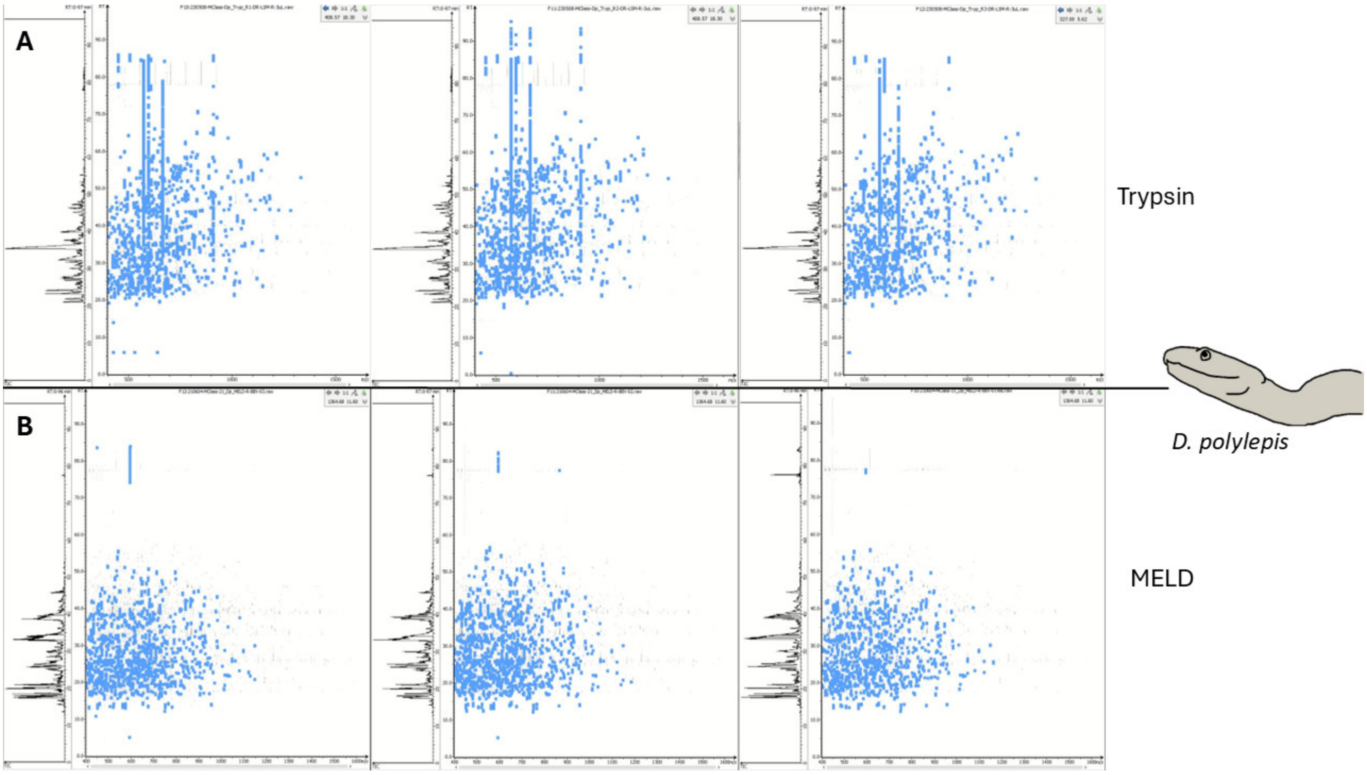

Figure S10. Peptide distribution plots from LC-MS analyses of *D. polylepis* venom samples. **(A)** Peptide mapping following trypsin-only digestion, in triplicate. **(B)** Peptide mapping obtained via MELD strategy, in triplicate. Each blue dot represents a peptide identified. The x-axis denotes the peptide mass-to-charge ratio (*m/z*), while the y-axis indicates the retention time (in minutes) during chromatographic separation. The unique peptides were used for relative quantification of toxin families in the tryptic digestion dataset.

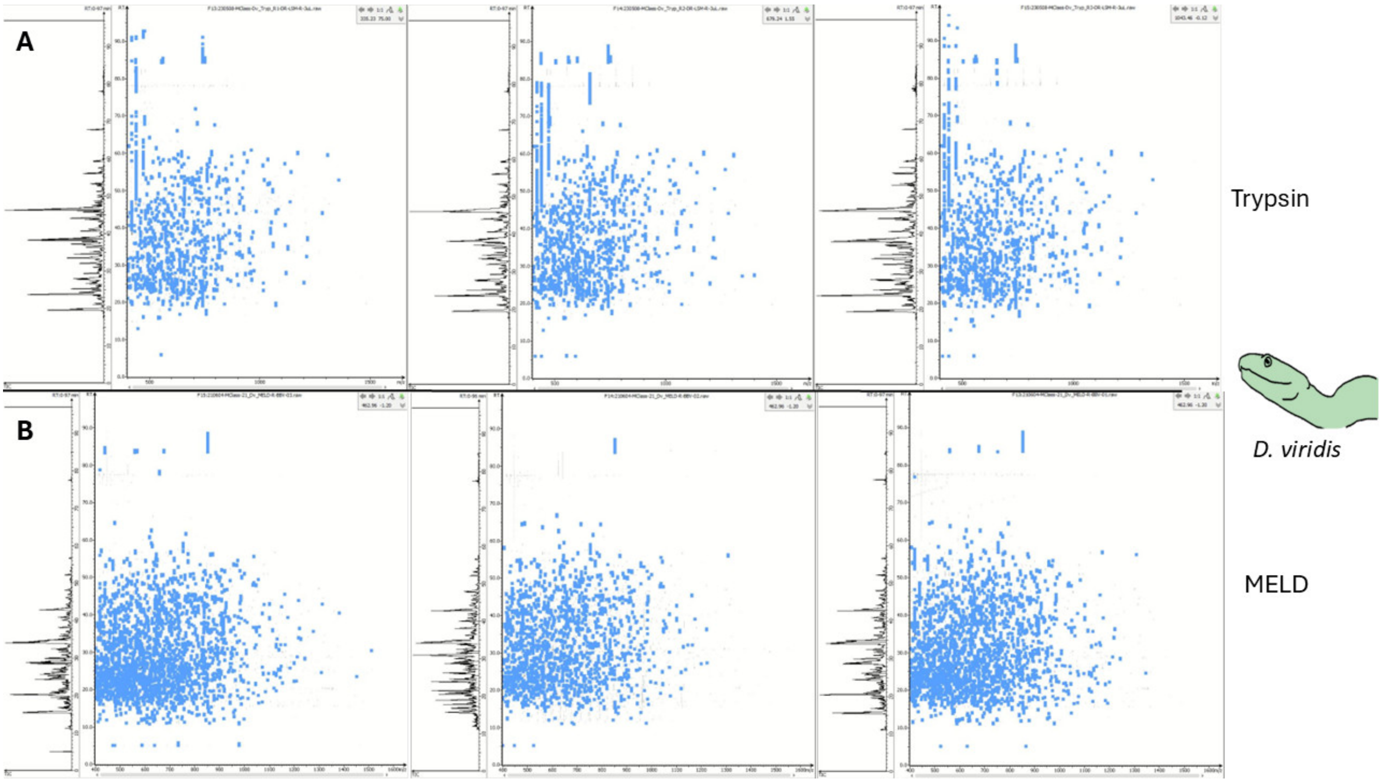

Figure S11. Peptide distribution plots from LC-MS analyses of *D. viridis* venom samples. **(A)** Peptide mapping following trypsin-only digestion, in triplicate. **(B)** Peptide mapping obtained via MELD strategy, in triplicate. Each blue dot represents a peptide identified. The x-axis denotes the peptide mass-to-charge ratio (*m/z*), while the y-axis indicates the retention time (in minutes) during chromatographic separation. The unique peptides were used for relative quantification of toxin families in the tryptic digestion dataset.

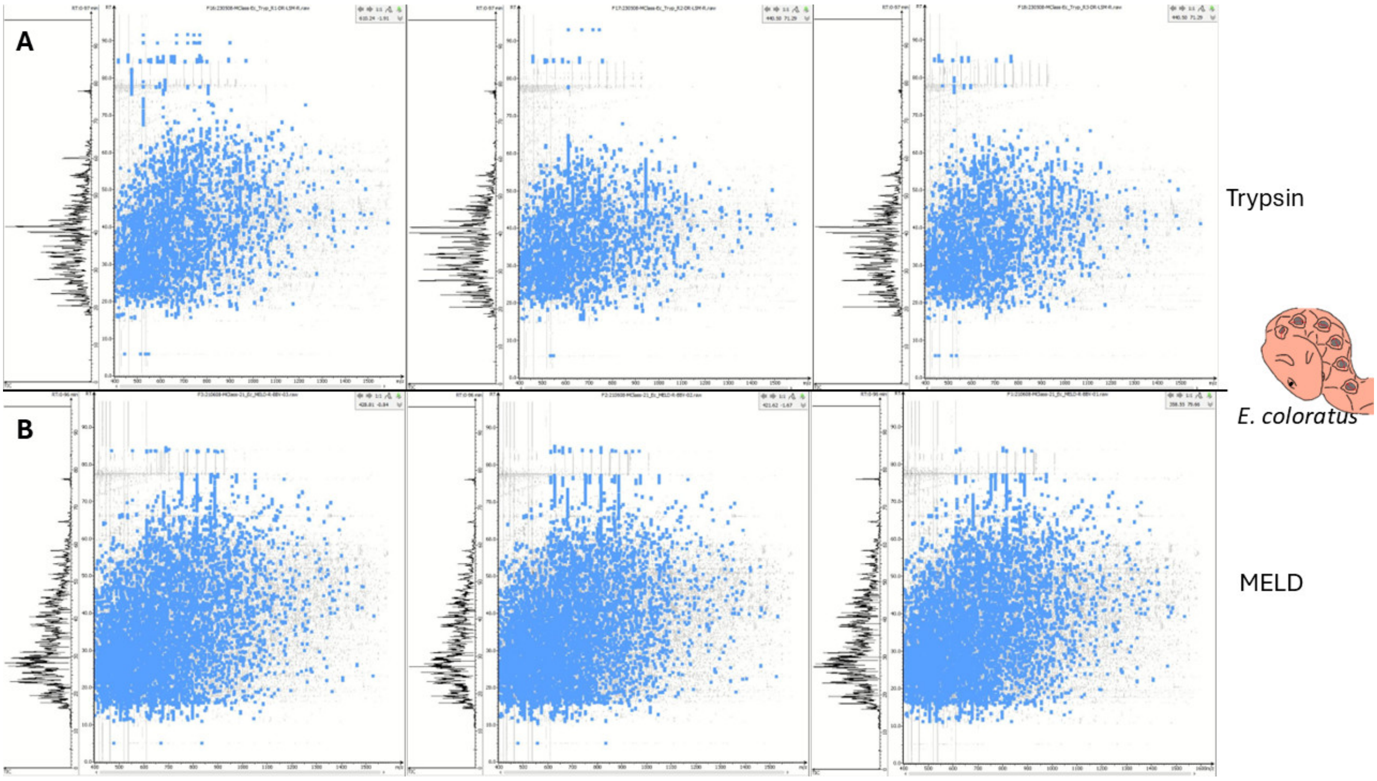

Figure S12. Peptide distribution plots from LC-MS analyses of *E. coloratus* venom samples. **(A)** Peptide mapping following trypsin-only digestion, in triplicate. **(B)** Peptide mapping obtained via MELD strategy, in triplicate. Each blue dot represents a peptide identified. The x-axis denotes the peptide mass-to-charge ratio (*m/z*), while the y-axis indicates the retention time (in minutes) during chromatographic separation. The unique peptides were used for relative quantification of toxin families in the tryptic digestion dataset.

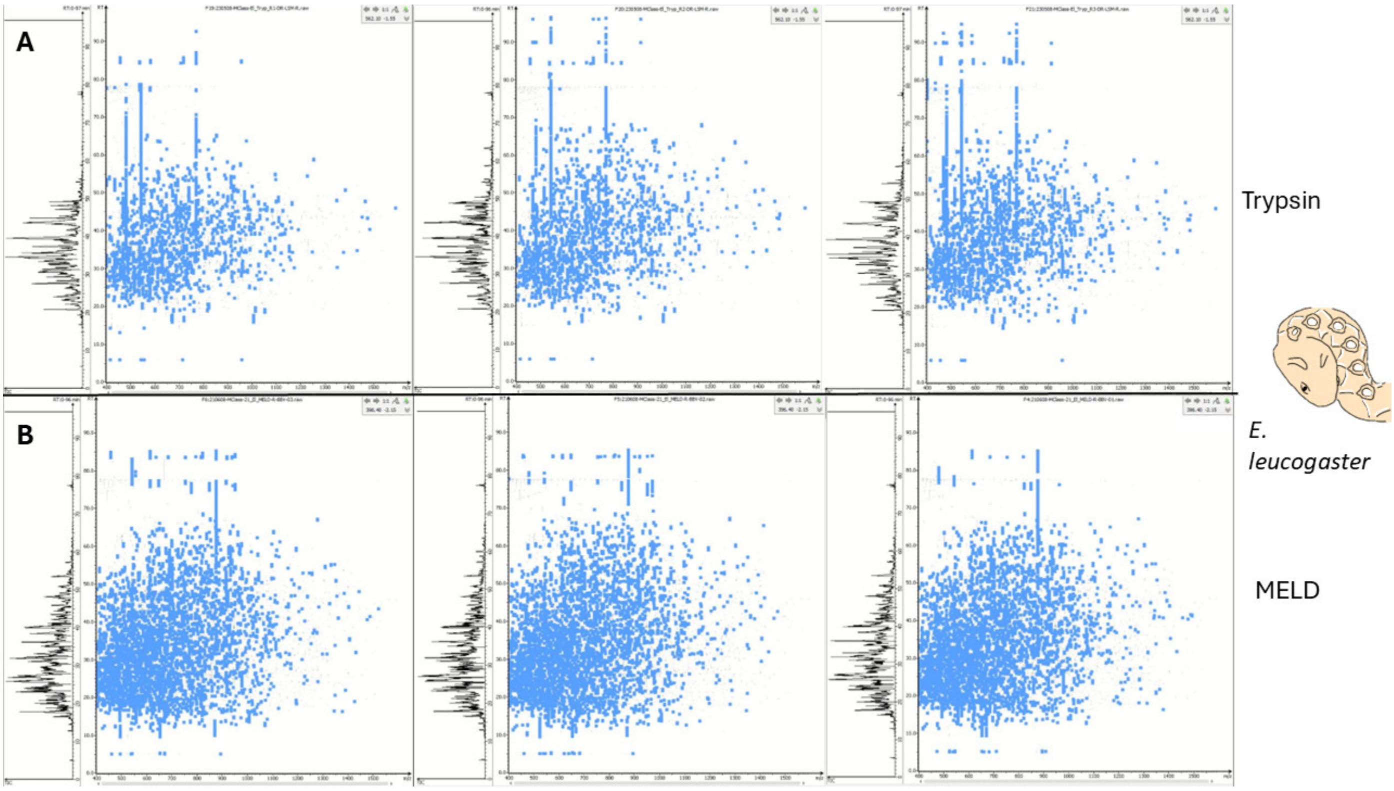

Figure S13. Peptide distribution plots from LC-MS analyses of *E. leucogaster* venom samples. **(A)** Peptide mapping following trypsin-only digestion, in triplicate. **(B)** Peptide mapping obtained via MELD strategy, in triplicate. Each blue dot represents a peptide identified. The x-axis denotes the peptide mass-to-charge ratio (*m/z*), while the y-axis indicates the retention time (in minutes) during chromatographic separation. The unique peptides were used for relative quantification of toxin families in the tryptic digestion dataset.

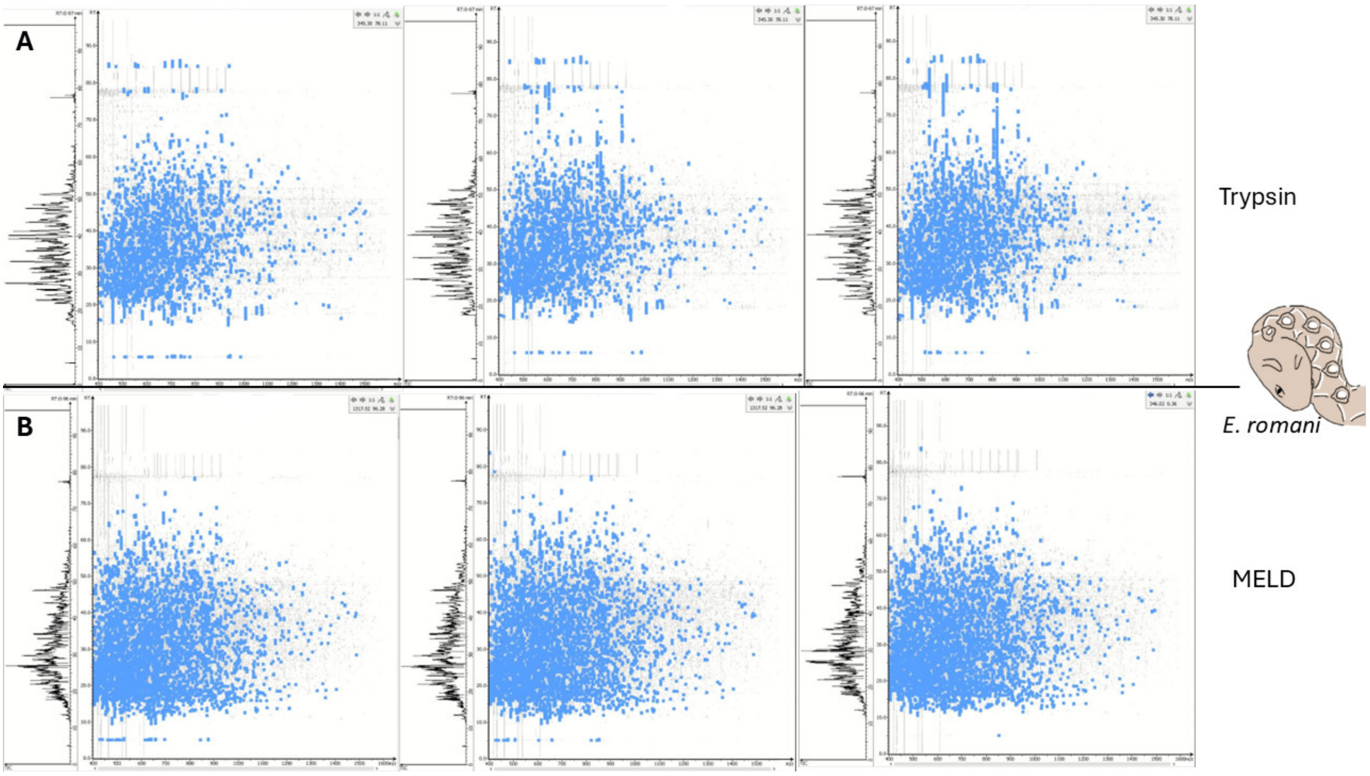

Figure S14. Peptide distribution plots from LC-MS analyses of *E. romani* venom samples. **(A)** Peptide mapping following trypsin-only digestion, in triplicate. **(B)** Peptide mapping obtained via MELD strategy, in triplicate. Each blue dot represents a peptide identified. The x-axis denotes the peptide mass-to-charge ratio (*m/z*), while the y-axis indicates the retention time (in minutes) during chromatographic separation. The unique peptides were used for relative quantification of toxin families in the tryptic digestion dataset.

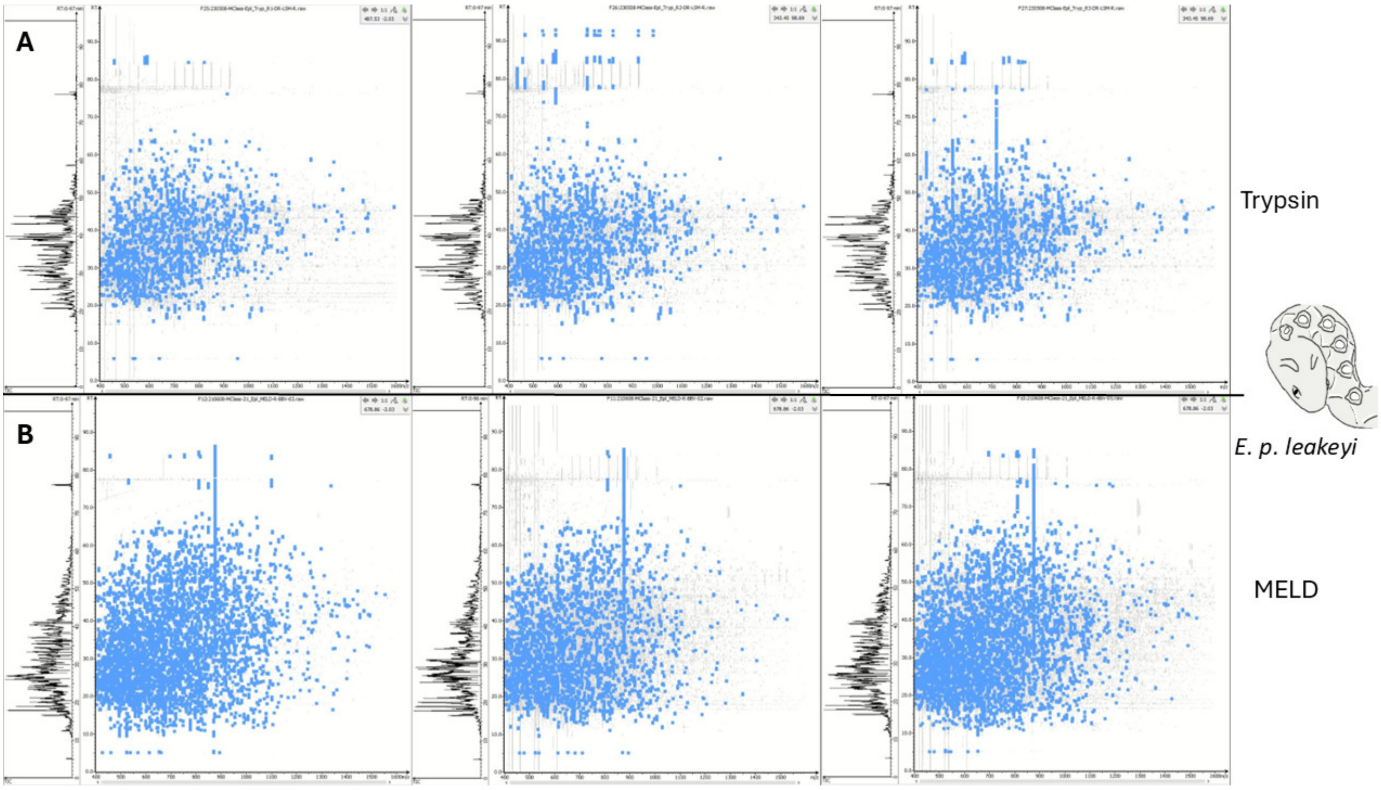

Figure S15. Peptide distribution plots from LC-MS analyses of *E. p. leakeyi* venom samples. **(A)** Peptide mapping following trypsin-only digestion, in triplicate. **(B)** Peptide mapping obtained via MELD strategy, in triplicate. Each blue dot represents a peptide identified. The x-axis denotes the peptide mass-to-charge ratio (*m/z*), while the y-axis indicates the retention time (in minutes) during chromatographic separation. The unique peptides were used for relative quantification of toxin families in the tryptic digestion dataset.
