# Supplementary figures and images for: Dual Proteomics Strategies to Dissect and Quantify the Components of Nine Medically Important African Snake Venoms
Source: Toxins (Basel). 2025 May 13;17(5):243. doi: 10.3390/toxins17050243 (PMC12116074; doi:10.3390/toxins17050243)

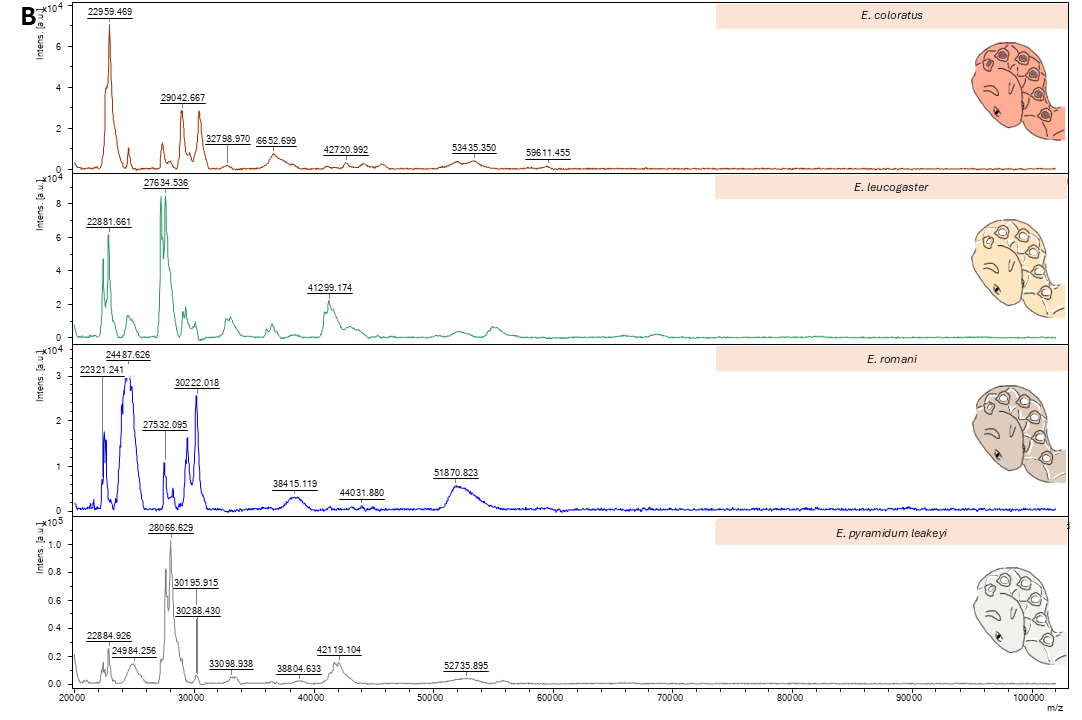

Supplement: Supplementary file 1 [file toxins-17-00243-s001.zip › Figure S6B.PNG]

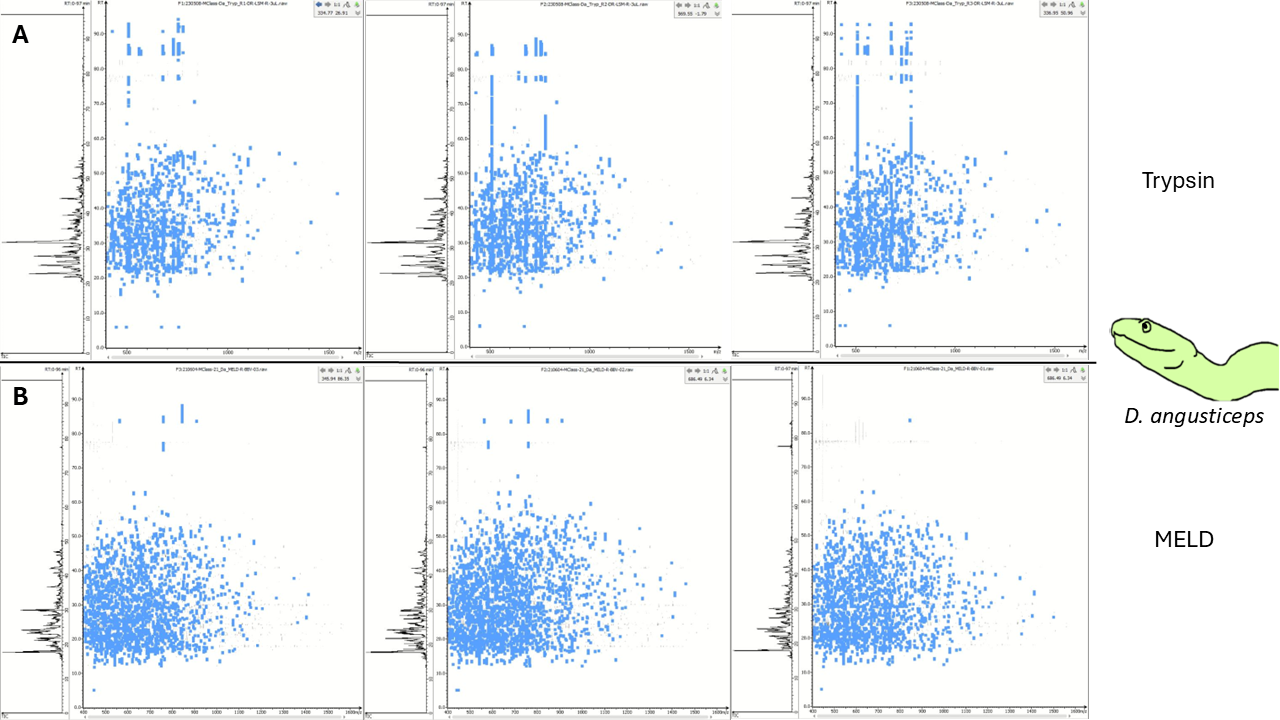

Supplement: Supplementary file 1 [file toxins-17-00243-s001.zip › Figure S7.PNG]

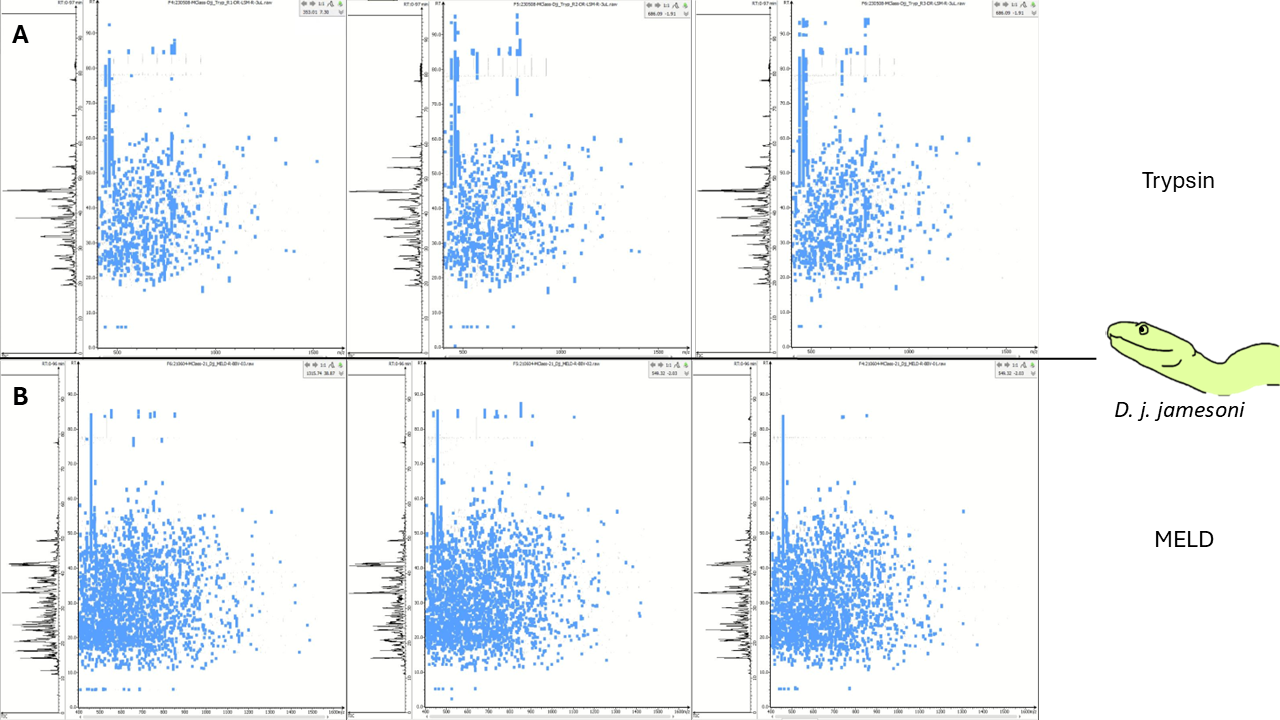

Supplement: Supplementary file 1 [file toxins-17-00243-s001.zip › Figure S8.PNG]

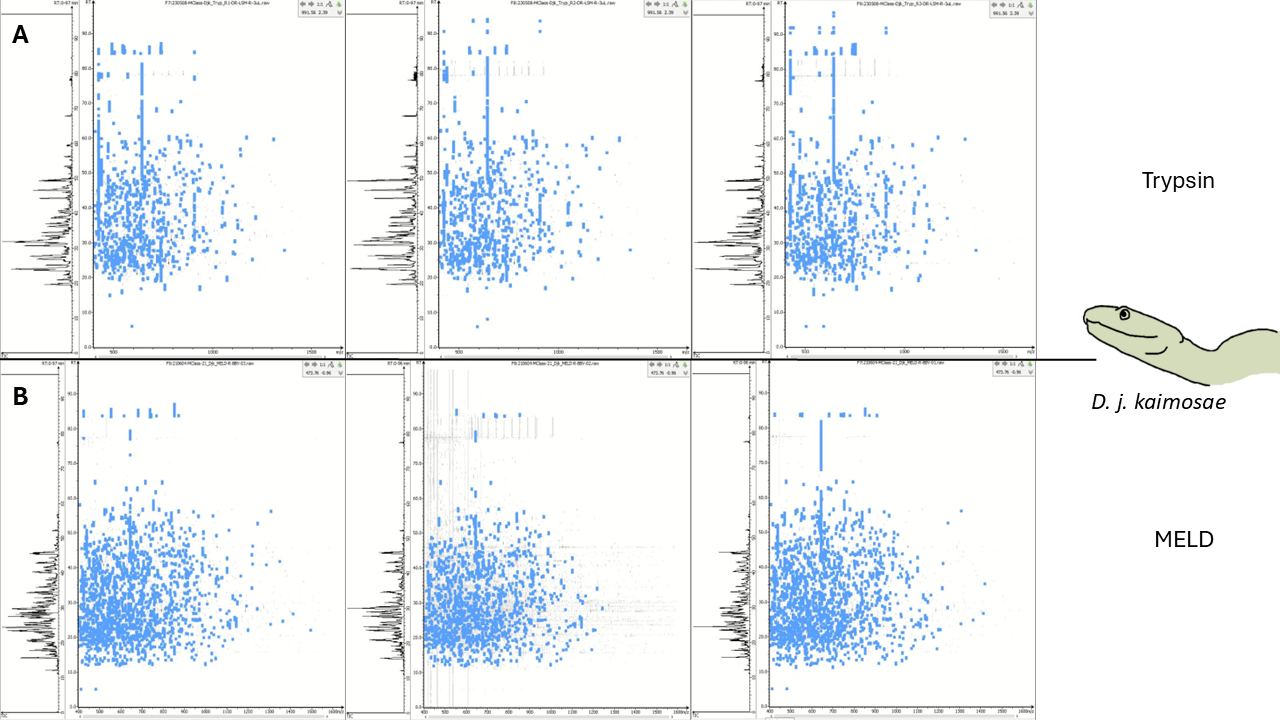

Supplement: Supplementary file 1 [file toxins-17-00243-s001.zip › Figure S9.PNG]

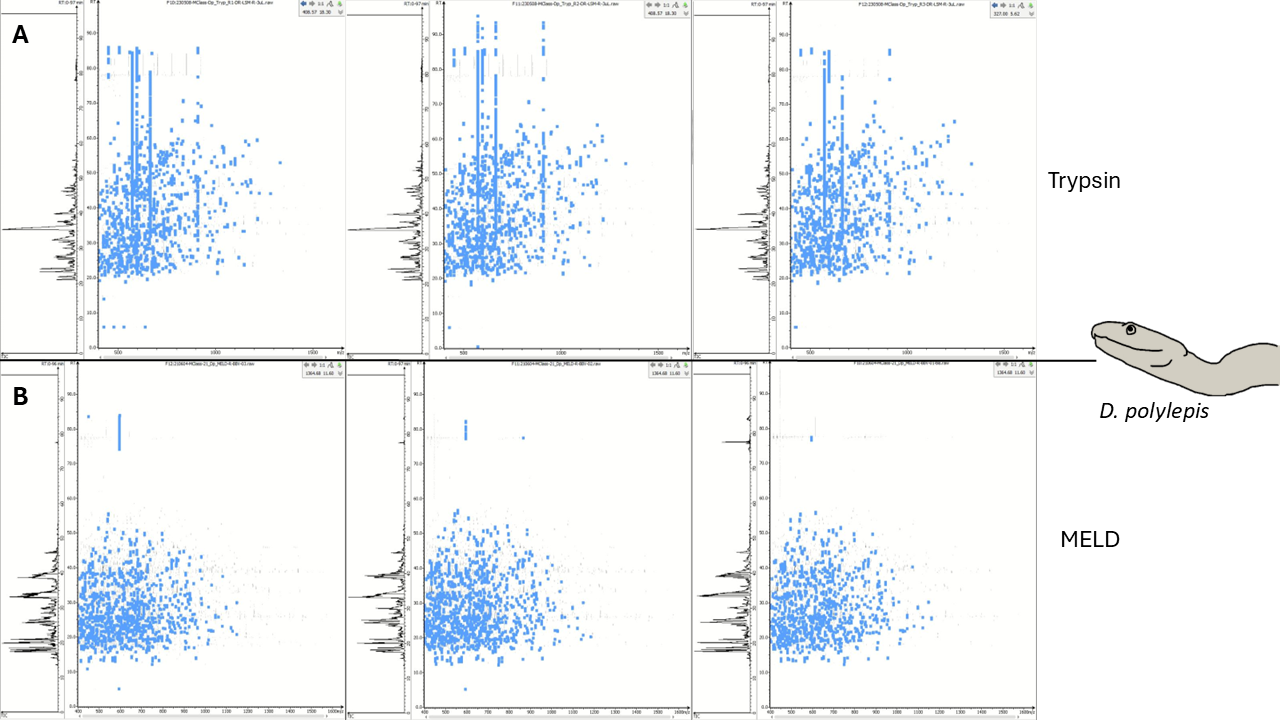

Supplement: Supplementary file 1 [file toxins-17-00243-s001.zip › Figure S10.PNG]

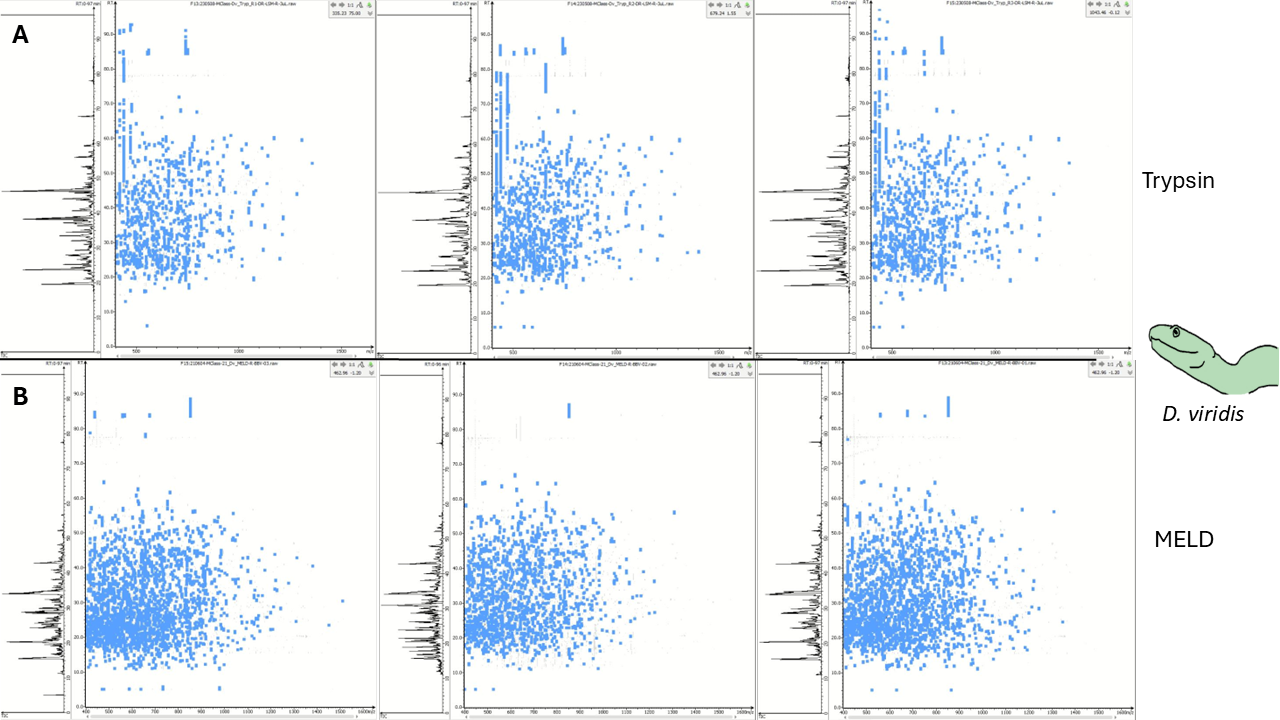

Supplement: Supplementary file 1 [file toxins-17-00243-s001.zip › Figure S11.PNG]

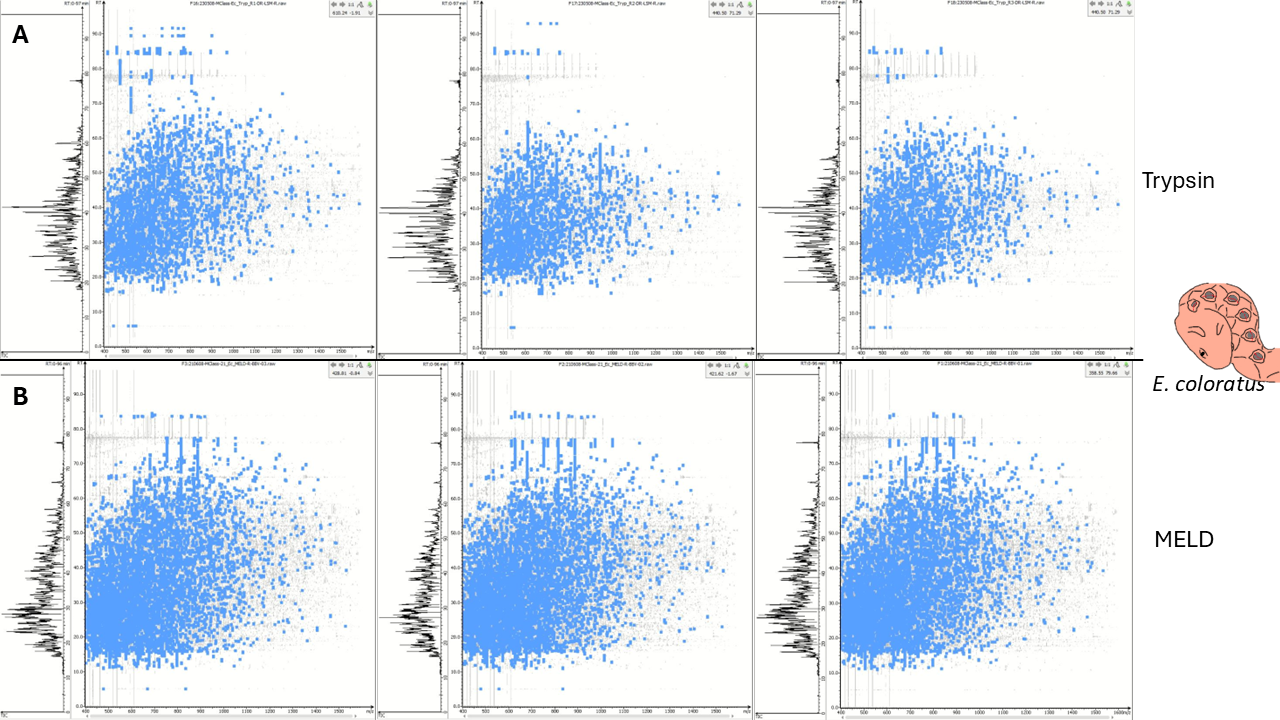

Supplement: Supplementary file 1 [file toxins-17-00243-s001.zip › Figure S12.PNG]

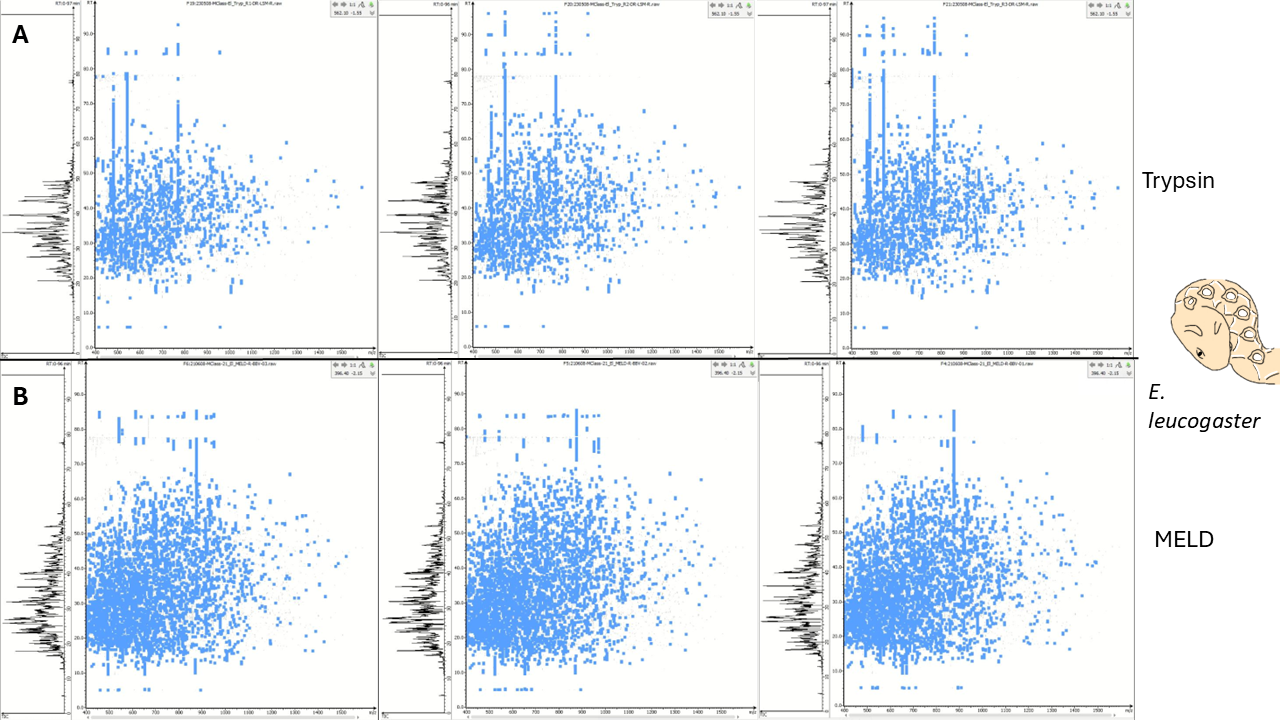

Supplement: Supplementary file 1 [file toxins-17-00243-s001.zip › Figure S13.PNG]

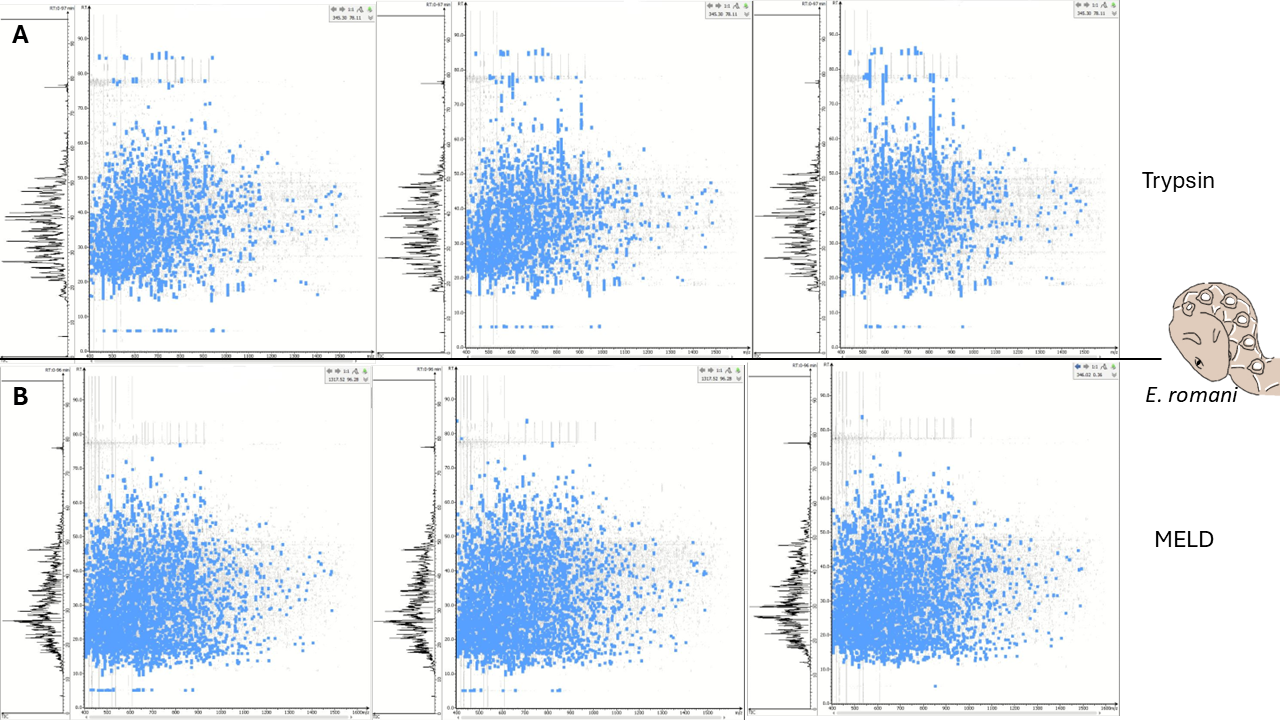

Supplement: Supplementary file 1 [file toxins-17-00243-s001.zip › Figure S14.PNG]

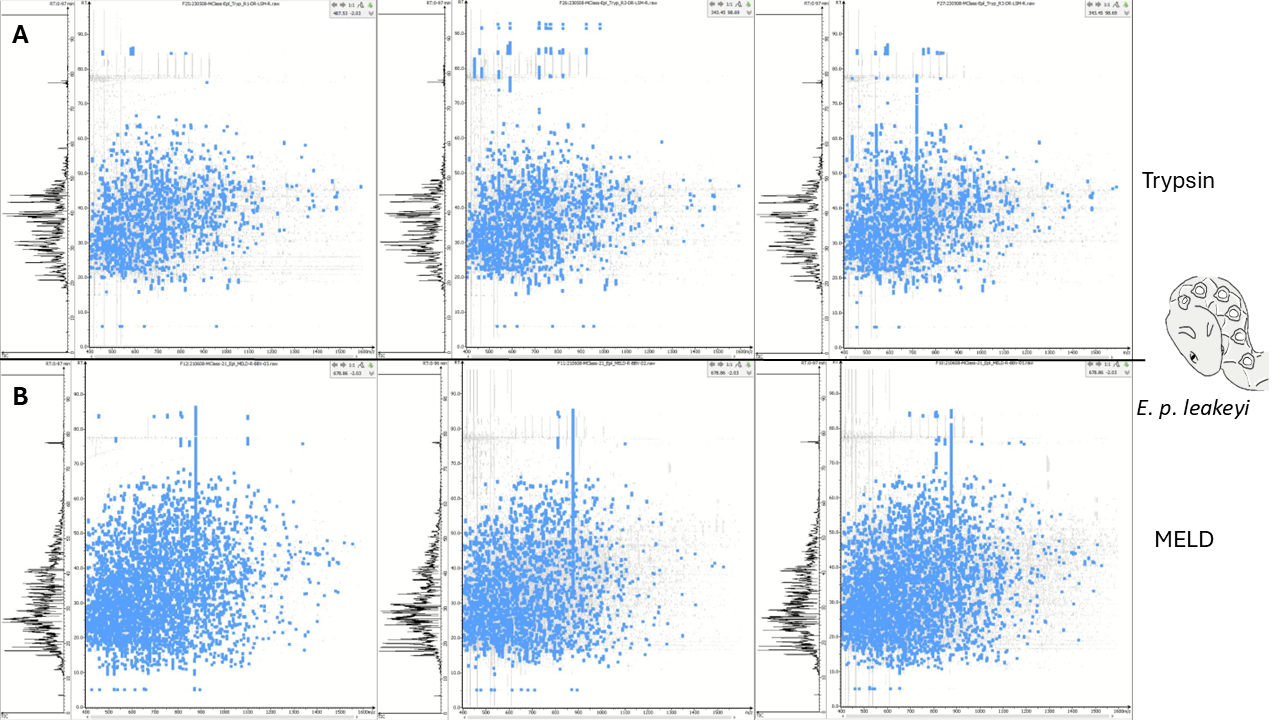

Supplement: Supplementary file 1 [file toxins-17-00243-s001.zip › Figure S15.PNG]

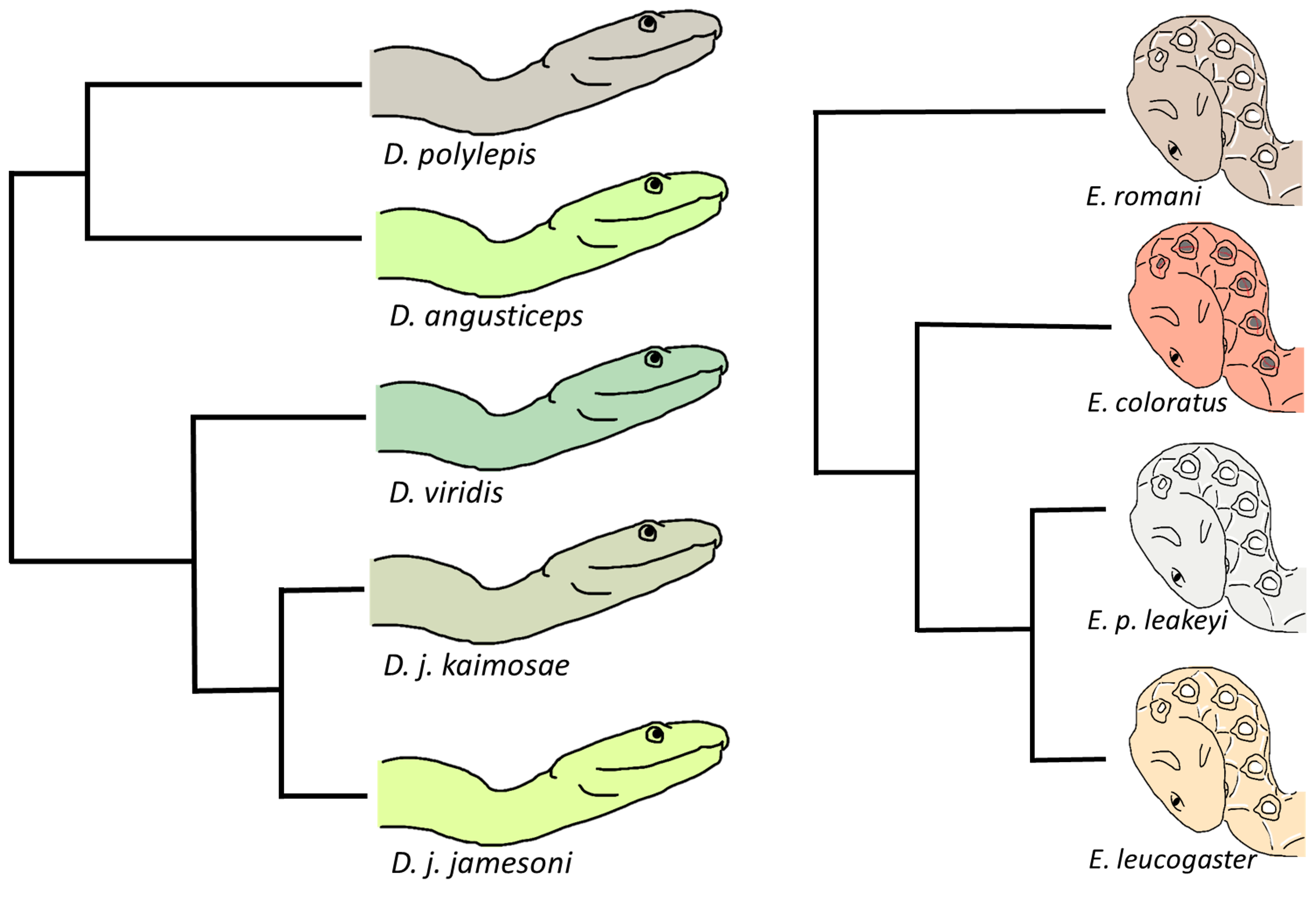

Supplement: Supplementary file 1 [file toxins-17-00243-s001.zip › Figure S1.png]

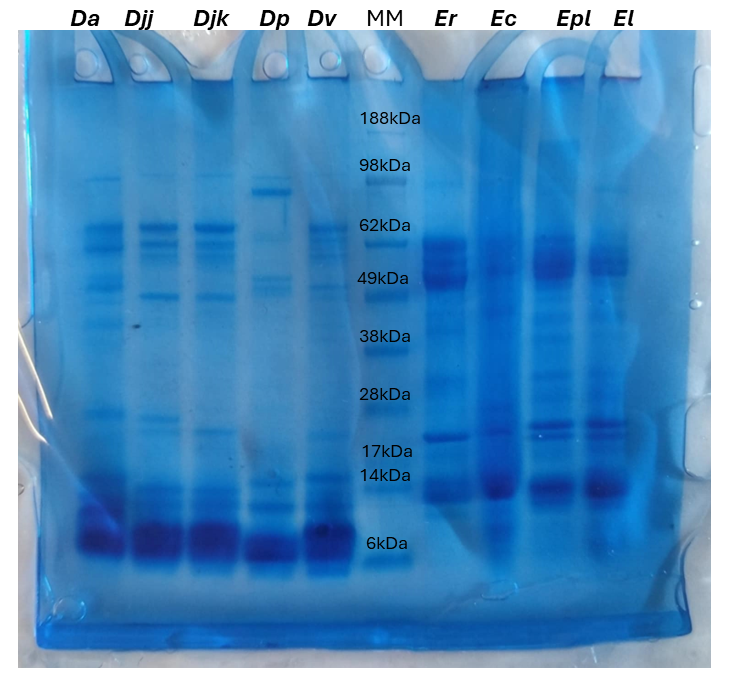

Supplement: Supplementary file 1 [file toxins-17-00243-s001.zip › Figure S2.PNG]

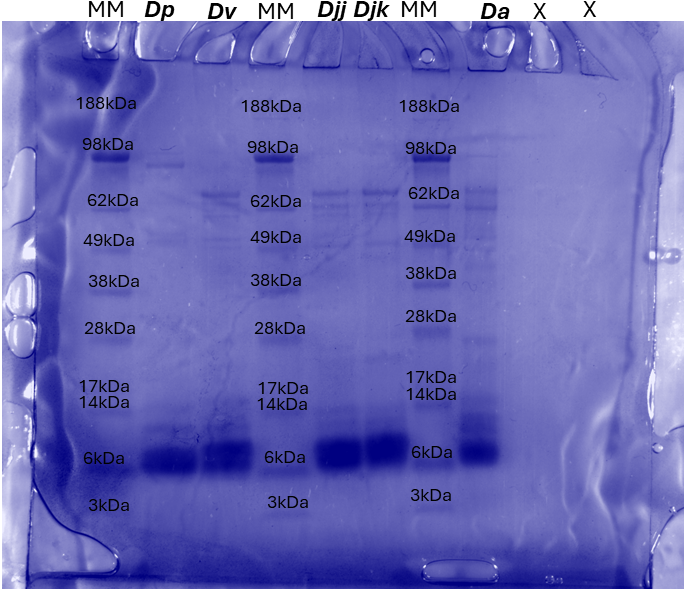

Supplement: Supplementary file 1 [file toxins-17-00243-s001.zip › Figure S3.PNG]

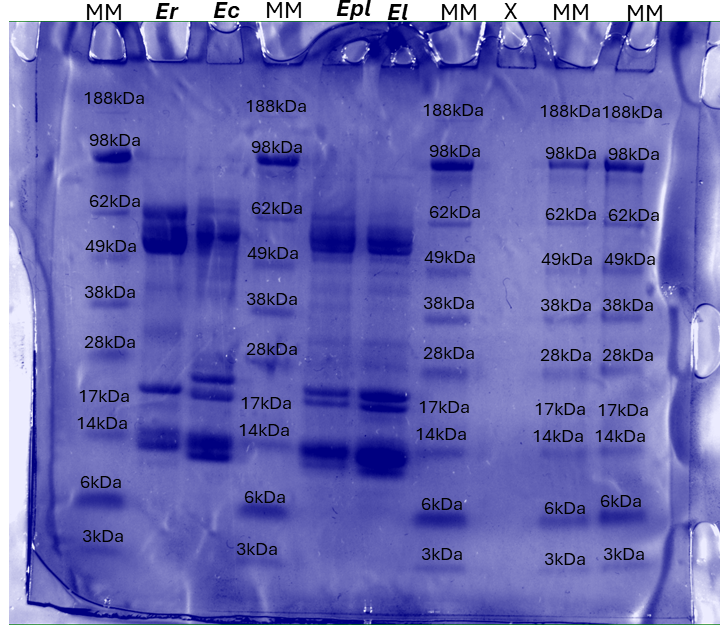

Supplement: Supplementary file 1 [file toxins-17-00243-s001.zip › Figure S4.PNG]

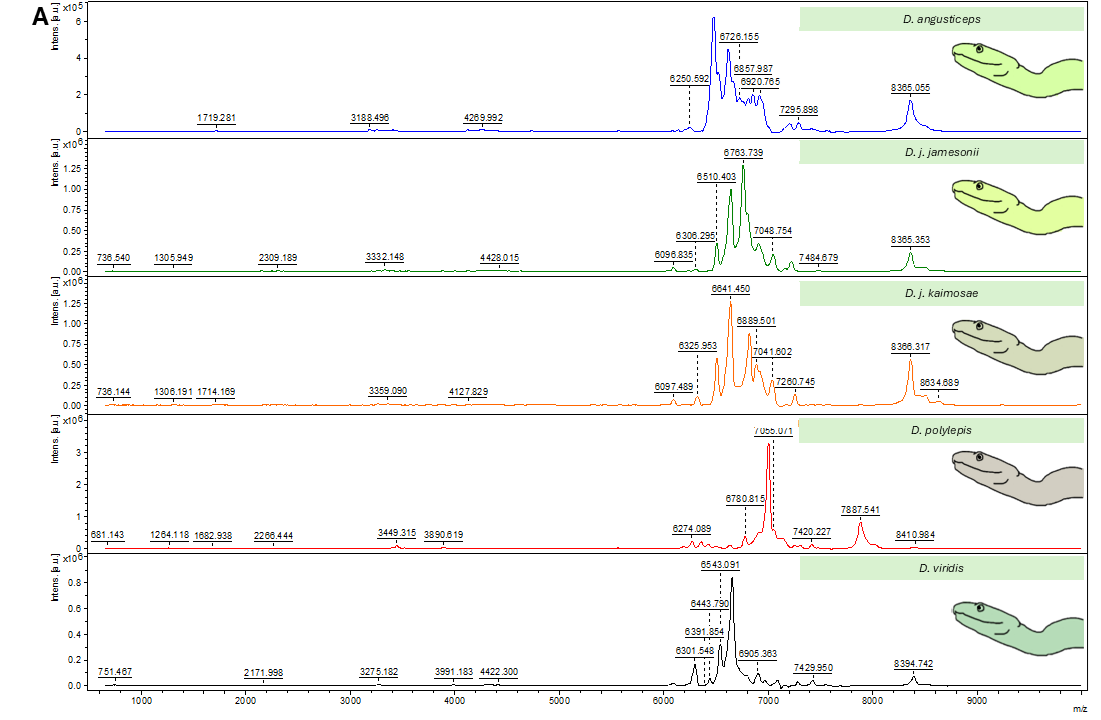

Supplement: Supplementary file 1 [file toxins-17-00243-s001.zip › Figure S5A.PNG]

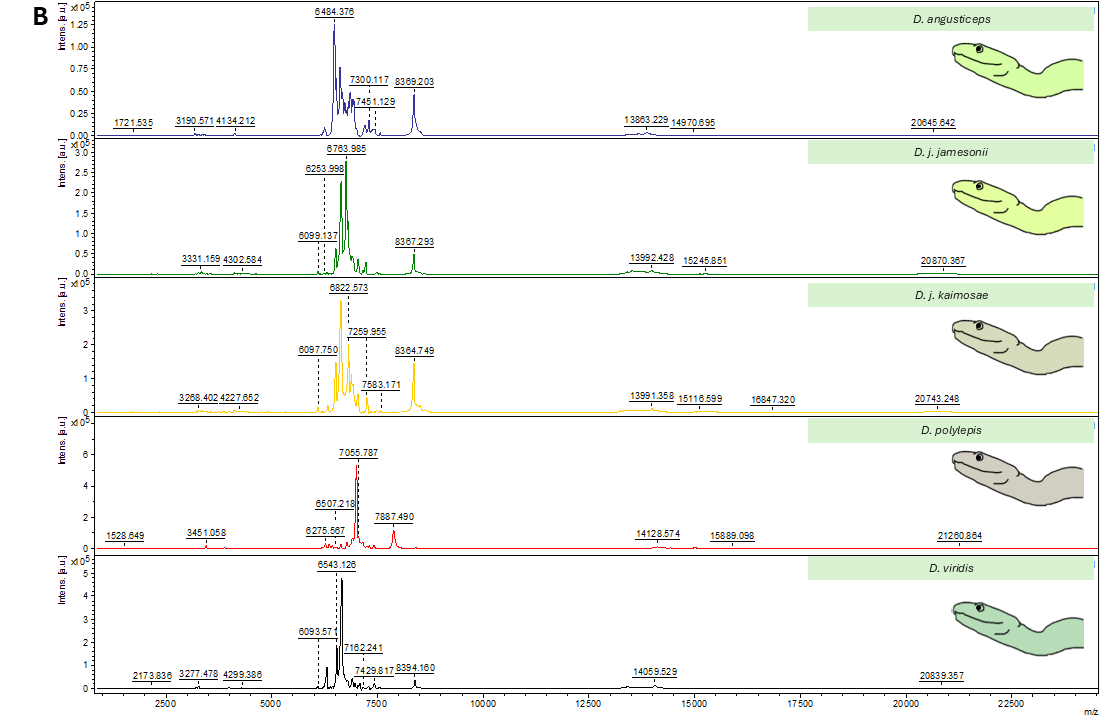

Supplement: Supplementary file 1 [file toxins-17-00243-s001.zip › Figure S5B.PNG]

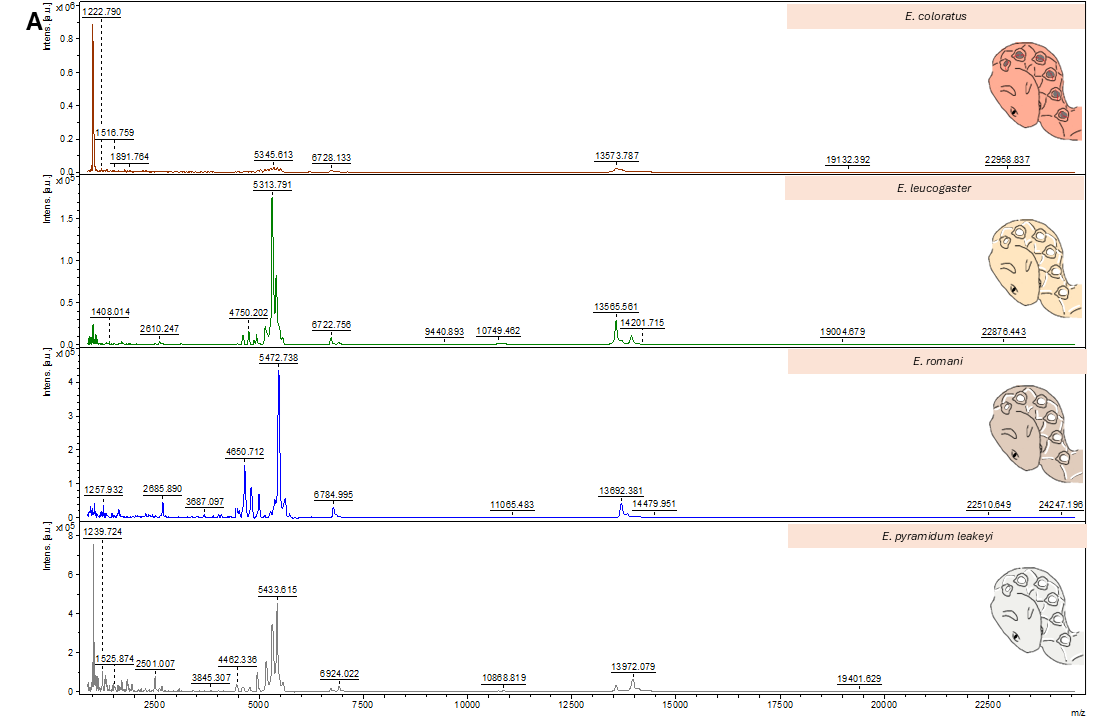

Supplement: Supplementary file 1 [file toxins-17-00243-s001.zip › Figure S6A.PNG]
